# Polar Constituents of *Salvia willeana* (Holmboe) Hedge, Growing Wild in Cyprus

**DOI:** 10.3390/plants7010018

**Published:** 2018-03-06

**Authors:** Theofilos Mailis, Helen Skaltsa

**Affiliations:** Department of Pharmacognosy & Chemistry of Natural Products, School of Pharmacy, National and Kapodistrian University of Athens, Panepistimiopolis, Zografou, 157 71 Athens, Greece; skaltsa@pharm.uoa.gr

**Keywords:** *Salvia willeana*, 4-hydroxy-acetophenone 4-*O*-(3,5-dimethoxy-4-hydroxybenzoyl)-*β*-d-glucopyranoside, megastigmane glucoside, phenolics, terpenes, 2D NMR, *Salvia* L: chemical review

## Abstract

Twenty compounds were isolated from the aerial parts of *Salvia willeana* (Holmboe) Hedge, growing wild in Cyprus. These compounds comprise one new and one known acetophenone, one megastigmane glucoside, five phenolic derivatives, two caffeic acid oligomers, three flavonoids, two lignans, two triterpene acids, one monoterpene glucoside, and two fatty acids. The structures of the isolated compounds were established by means of NMR [(Rotating-frame OverhauserEffect SpectroscopY) (^1^H-^1^H-COSY (COrrelation SpectroscopΥ), ^1^H-^13^C-HSQC (Heteronuclear Single Quantum Correlation), HMBC (Heteronuclear Multiple Bond Correlation), NOESY (Nuclear Overhauser Effect SpectroscopY), ROESY (Rotating-frame Overhauser Effect SpectroscopY)] and MS spectral analyses. This is the first report of the natural occurrence of 4-hydroxy-acetophenone 4-*O*-(3,5-dimethoxy-4-hydroxybenzoyl)-*β*-d-glucopyranoside. A chemical review on the non-volatile secondary metabolites has been carried out. Based on the literature data, the analysis revealed that the chemical profile of *S. willeana* is close to that of *S. officinalis* L.

## 1. Introduction

The Lamiaceae family consists of more than 250 genera; *Salvia* L. is the largest genus within this family due to the presence of approximately 900 species. *Salvia* L. spreads in the warm and temperate regions of both the northern and southern hemispheres, and some species of this genus have been cultivated worldwide for use in folk medicine, in perfumery and cosmetics industries, as well as for culinary purposes, like flavoring and aromatic agents [1,2]. Some of its many interesting biological and pharmacological properties are its antioxidant [3], antimicrobial [3,4], cytotoxic [3,4], anti-HIV [3], and antiplasmodial effects [4], as well as others [3]. It is noteworthy that the name of the genus, *Salvia*, is derived from the Latin word “salvare”, which means “to save”, in reference to the curative properties of the plant [5]. The genus has attracted such great interest, that it has become the subject of numerous chemical studies, giving evidence that these plants are a rich source of a wide variety of secondary metabolites, such as polyphenols and terpenoids [1].

*Salvia willeana* (syn. *S. grandiflora* subsp. *willeana* Holmboe and *S. grandiflora* subsp. *albiflora* Lindb.) is a low-growing, strongly aromatic suffruticose herb, sometimes carpeting the ground [6]. This species is endemic to Cyprus, where it grows on moist, rocky mountainsides of the Troodos range at 1150–1950 m altitude and it flowers from May to October [7]. Its extracts possess different pharmacological properties and the plant has been used to halt milk production in nursing mothers, as well as for its antiseptic activity [8]. As *S. willeana* is locally used in aqueous preparations, the purpose of our study was the investigation of secondary metabolites obtained from the polar extract of its aerial parts. Our previous study of its lipophilic extract, revealed the presence of camphor, lupeol, and oleanolic acid, and demonstrated their anti-inflammatory effect [8]. It is of interest to note that there is only one more report regarding the chemical constituents of a polar extract of *S. willeana*, which revealed the presence of the triterpenoids ursolic and oleanolic acids, the diterpenoids carnosic acid and isorosmanol, and the flavonoid salvigenin [9].

## 2. Results

**Figure 1:**
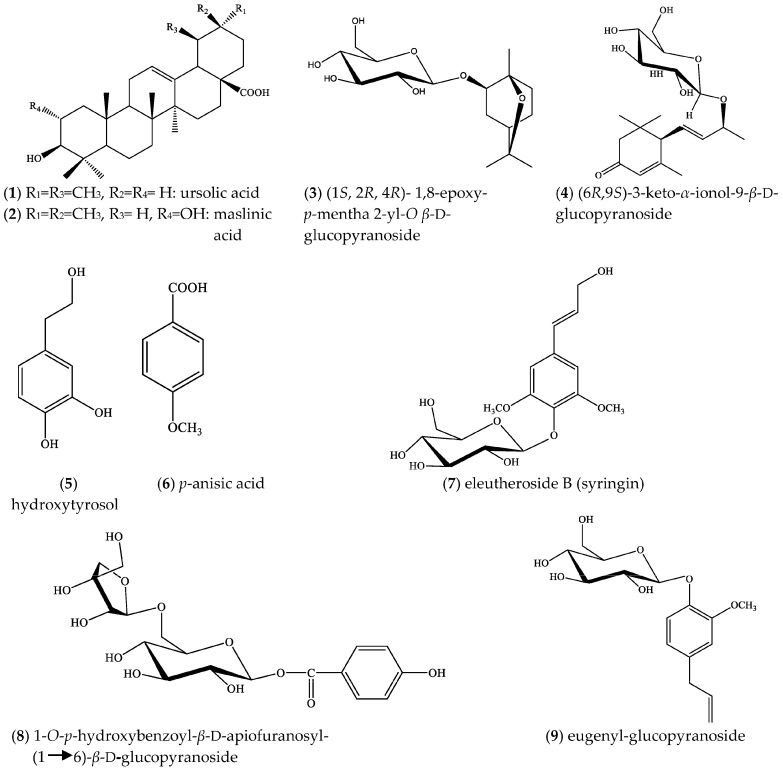

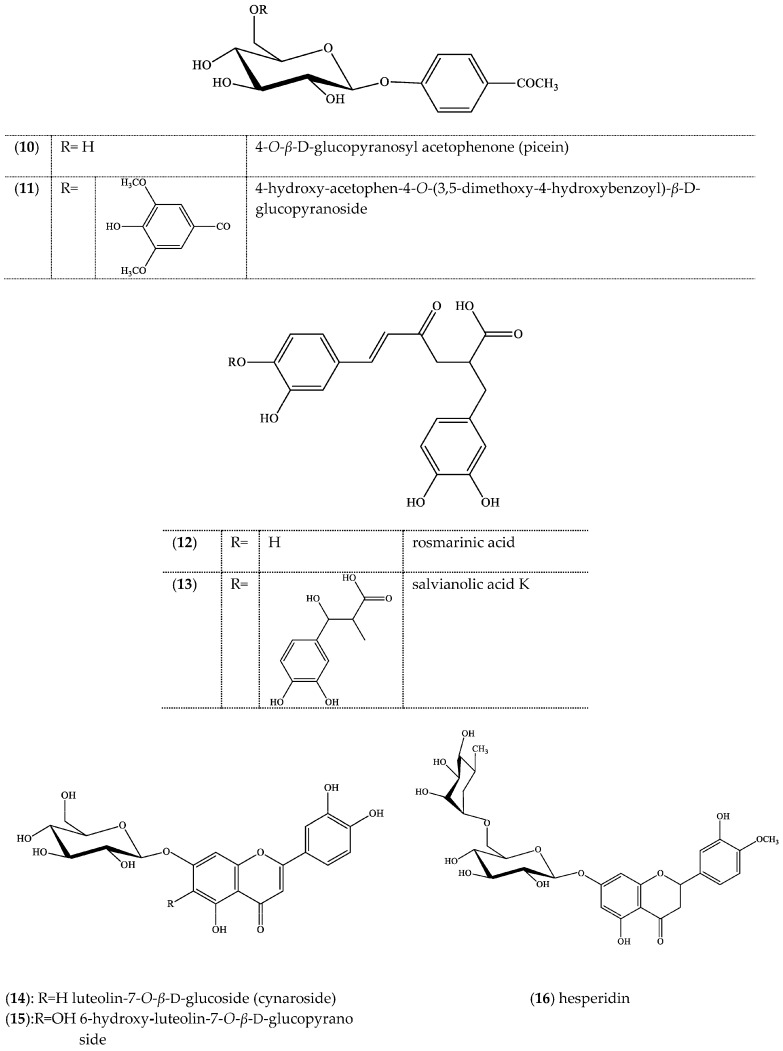

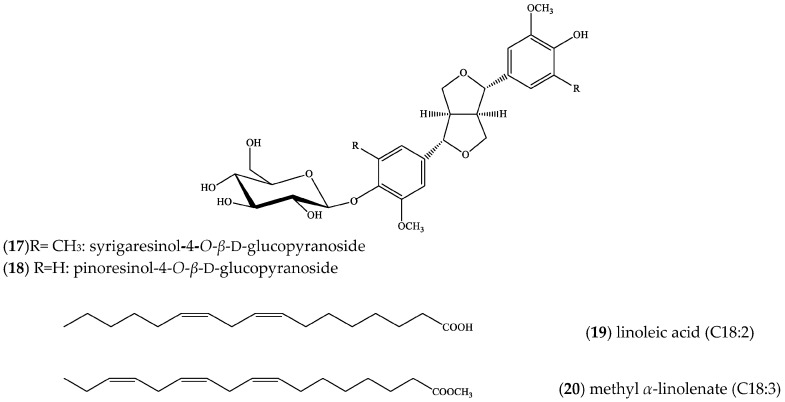
Structures of isolated compounds from *Salvia willeana.*

The polar extract (MeOH:H_2_O 5:1) of *S. willeana* was fractionated by MPLC (medium pressure liquid chromatography), CC (column chromatography), followed by semi-preparative HPLC, preparative TLC, and yielded two triterpenoids, namely ursolic acid (**1**) and maslinic acid (**2**), one monoterpene glucoside, (1*S*,2*R*,4*R*)-1,8-epoxy-*p*-menthan-2-yl-*O-β*-d-glucopyranoside (**3**), one megastigmane glucoside, (6*R*,9*S*)-3-oxo-*α*-ionol *β*-d-glucopyranoside (**4**), five phenolic compounds, i.e., hydroxy-tyrosol (**5**), *p*-anisic acid (**6**), eleutheroside B (syringin) (**7**), 1-*O*-*p*-hydroxybenzoyl-*β*-d-apiofuranosyl-(1→6)-*β*-d-glucopyranoside (**8**) and eugenylglucoside (**9**), two acetophenones, 4-*O*-*β*-d-glucopyranosyl acetophenone (picein) (**10**) and 4-hydroxy-acetophenone 4-*O*-(3,5-dimethoxy-4-hydroxybenzoyl)-*β*-d-glucopyranoside (**11**), two caffeic acid oligomers, rosmarinic acid (**12**) and salvianolic acid K (**13**), three flavonoids, luteolin-7-*O*-*β*-d-glucoside (cynaroside) (**14**), 6-hydroxyluteolin 7-*O*-*β*-d-glucoside (**15**) and hesperidin (**16**), two lignans, syringaresinol-4-*O*-*β*-d-glucopyranoside (**17**), pinoresinol-4-*O*-*β*-d-glucopyranoside (**18**), and two fatty acids: linoleic acid (**19**) and methyl *α*-linolenate (**20**) (Figure 1).

**Figure 2:**
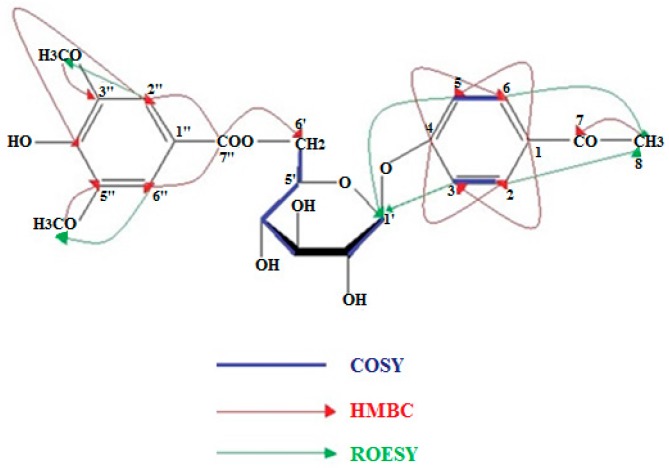
COSY, HMBC, and ROE signals of compund **11.**

Compound **11** was obtained as a white amorphous powder. ${\left[a\right]}_{D}^{20}$ − 4.71° (c 0.25 MeOH); UV (CH_3_OH) *λ*_max_: 272 nm. The HREIMS of **11** established its molecular formula as C_23_H_26_O_11_ (found 477.1405 [M − H]^−^, calcd. 478.1449). The ^1^H-NMR spectrum (Table 1) showed signals at *δ*_H_ 7.69 (2H, d, *J* = 9.0 Hz) and 7.05 (2H, d, *J* = 9.0 Hz), which were indicative of a 1,4-bisubsituted phenyl group. Thus, these proton signals were recognized belonging to the aromatic ring of the acetophenone moiety [10]. In the upfield region of the ^1^H-NMR spectrum, a singlet at *δ*_H_ 2.47 (3H, s) was ascribed to the methyl group (CH_3_-8) attached on the carbonyl group of the acetophenone [10]. Moreover, the presence of a singlet at *δ*_H_ 7.33 with an integration of two aromatic protons, indicative of a pair of equivalent methine protons, revealed the occurrence of the syringic acid ester structure in the molecule [11]. In the ^1^H-NMR spectrum, the presence of a *β*-d-glucopyranose unit was evident based on a characteristic doublet signal with a coupling constant of 7.9 Hz at *δ*_H_ 5.04, assignable to the anomeric proton of the sugar moiety. Moreover, in the ^1^H-^1^H-COSY spectrum the correlation peaks between the vicinal protons of the sugar ring were observed. Furthermore, from the ^13^C-NMR data (Table 1) the carbon signals of the glucose moiety were assigned at *δ*_C_ 101.7 (C-1′), 77.5 (C-3′), 75.5 (C-5′), 74.5 (C-2′), 71.3 (C-4′), and 64.8 (C-6′), matching the reported data of 1,6-disubstituted-*β*-d-glucose [12]. The structural assignment was further confirmed by HSQC experiments, due to the carbon signals at *δ*_C_ 130.1 (C-2/C-6), 116.2 (C-3/C-5), and 26.5 (C-8), indicative of a 4-hydroxy-acetophenone moiety [9,13], while the syringic acid ester was confirmed by the presence of the carbon signals at *δ*_C_ 108.3 (C-2″/C-6″), 56.0 (3″, 5″-OCH_3_) [11]. The existence of the acetophenone moiety was corroborated by the HMBC experiment. This spectrum revealed a long-range cross peak between the carbonyl group at *δ*_C_ 199.1 (C-7) with the equivalent protons H-2/H-6 (*δ*_H_ 7.69), as well as an interaction between the carbonyl group (*δ*_C_ 199.1) and the methyl group CH_3_-8 (*δ*_H_ 2.47). In addition, the linkage of the syringyl moiety with the glucosyl moiety was substantiated by the observation of an HMBC correlation between the carbonyl group at *δ*_C_ 167.3 and the 6′-methylene protons at *δ*_H_ 4.46 (dd, *J* = 11.7, 8.0). Furthermore, the position of the attachment of the carboxyl group to the quaternary carbon C-1″ of the syringic ester was determined by a diagnostic HMBC cross peak between the equivalent methine protons H-2″/H-6″ at *δ*_H_ 7.33 and the carbonyl carbon C-7″ at *δ*_C_ 167.3, while a long-range coupling between the methyl protons of the methoxy groups at *δ*_H_ 3.83 and the benzylic carbons C-3″/C-5″ at *δ*_C_ 149.0 was also observed. Moreover, the position of the attachment of glucose to the 4-hydroxy-acetophenone moiety was revealed by a ROESY experiment, which displayed correlations between the anomeric proton H-1′ (*δ*_H_ 5.04) and the equivalent protons H-3/H-5 (*δ*_H_ 7.05) of the acetophenone (Figure 2). On the basis of the information above and by comparison with the data for compounds of similar structures [10,11,13] compound **11** was identified as 4-hydroxy-acetophenone 4-*O*-(3,5-dimethoxy-4-hydroxybenzoyl)-*β*-d-glucopyranoside, which is a new natural product, to the best of our knowledge.

The identification of the known flavonoids luteolin-7-*O*-*β*-d-glucoside (**14**) [14,15,16], 6-hydroxyluteolin 7-*O*-*β*-d-glucoside (**15**) [17], and hesperidin (**16**) [18,19,20,21,22,23,24] was based on UV–VIS and NMR spectroscopic analyses, as well as by comparing their spectroscopic data with those reported in the literature. The structure of the two fatty acids, linoleic acid (**19**) [25,26] and methyl *α*-linolenate (**20**) [26,27], has been deduced by the interpretation of NMR and GC-MS data. The ^1^H-NMR chemical shifts for compounds **1** [28,29,30], **2** [31,32,33], **3** [34,35], **4** [35,36,37], **7** [38,39,40,41,42], **9** [35,43], **10** [10,35,44], **12** [35,45,46,47,48], **17** [49,50], and **18** [51,52,53,54] presented in our study are in agreement with the data previously reported in the literature.

However, the NMR data of compounds **5**, **6**, **8**, and **13** are not fully recorded in the literature, therefore, they are presented here below.

## 3. Discussion

The genus *Salvia* L. is characterized by the presence of several different secondary metabolites, mainly phenolic derivatives and terpenoids [3].

In the present study, overall, 20 compounds were isolated from *S. willeana* polar extracts, i.e., two triterpenoids, namely ursolic acid (**1**) and maslinic acid (**2**), one monoterpene glucoside, (1*S*,2*R*,4*R*)-1,8-epoxy-*p*-menthan-2-yl-*O*-*β*-d-glucopyranoside (**3**), one megastigmane glucoside, (6*R*,9*S*)-3-oxo-*α*-ionol *β*-d-glucopyranoside (**4**), five simple phenolic compounds, i.e., hydroxy-tyrosol (**5**), *p*-anisic acid (**6**), eleutheroside B (syringin) (**7**), 1-*O*-*p*-hydroxybenzoyl-*β*-d-apiofuranosyl-(1→6)-*β*-d-glucopyranoside (**8**) and eugenylglucoside (**9**), two acetophenones, 4-*O*-*β*-d-glucopyranosyl acetophenone (picein) (**10**) and 4-hydroxy-acetophenone 4-*O*-(3,5-dimethoxy-4-hydroxybenzoyl)-*β*-d-glucopyranoside (**11**), two caffeic acid oligomers, rosmarinic acid (**12**) and salvianolic acid K (**13**), three flavonoids, luteolin-7-*O*-*β*-d-glucoside (cynaroside) (**14**), 6-hydroxyluteolin 7-*O*-*β*-d-glucoside (**15**) and hesperidin (**16**), two lignans, syringaresinol-4-*O*-*β*-d-glucopyranoside (**17**), pinoresinol-4-*O*-*β*-d-glucopyranoside (**18**) and two fatty acids: linoleic acid (**19**) and methyl *α*-linolenate (**20**). 

It is interesting to point out that compounds **7**, **16**, **17**, and **18** had not been previously detected in *Salvia* L. Syringin (**7**) is reported, here, as a component of the Lamiaceae family for the first time. Moreover, compounds **3**, **5**, **6**, and **8**–**10** had been previously mentioned only once in the genus, as follows: **3**, **9**, **10** [35], **6** [55], **8** [10] and **14**, **15** [3] in *S. officinalis* L., **5** in *S. digitaloides* Diels [56], while compound **4** twice in *S. nemorosa* and *S. officinalis* L. [35,37]. As for the two triterpenoids **1** and **2**, these have previously been isolated from the acetone extract of the aerial parts of *S. willeana* [9]. So far, only 134 *Salvia* species of the over 1000 species suggested have been investigated [3]. Based on our results, concerning the polar secondary metabolites, among these species *S. willeana* showed many similarities to *S. officinalis* L., since most of the isolated simple phenols (**5**, **6**, **8**–**10**), as well as the flavonoids **14** and **15**, are found only in these two species (Supplementary Materials Table S1 and the references herein).

## 4. Materials and Methods

### 4.1. Plant Material

Aerial parts of *Salvia willeana* (Holmboe) Hedge were collected on Troodos Mountain in Cyprus in April 2004 [8]. A voucher specimen has been deposited in the Agricultural Research Institute Herbarium of Nicosia [no. ARI 3213].

### 4.2. Equipment and Reagents

^1^H, ^13^C, and 2D-NMR spectra were recorded in CDCl_3_ and CD_3_OD on Bruker DRX 400 (399.95 MHz for ^1^H-NMR) and Bruker AC 200 (200.13 MHz for ^1^H-NMR and 50.3 MHz for ^13^C-ΝMR) instruments at 295 K (Bruker BioSpin GmbH, Silberstetten, Germany). Chemical shifts are given in ppm (*δ*) and were referenced to the solvent signals at 7.24/3.31 and 77.0/49.5 ppm for ^1^H and ^13^C-NMR, respectively. COSY, HSQC, HMBC, NOESY, and ROESY (mixing time 950 ms) were performed using standard Bruker microprograms. High-resolution mass spectra were measured on a Q-TOF 6540 UHD (Aligent Technologies, Santa Clara, California, USA). The solvents used were of spectroscopic grade (Merck KGaA, Darmstadt, Germany). UV spectra were recorded on a Shimadzu UV-160A spectrophotometer (Shimadzu; Kyoto, Japan), according to Mabry et al. [57]. Optical rotations were determined using a Perkin-Elmer Polarimeter 341 (Perkin-Elmer, GmbH, Überlingen, Germany). GC-MS (Gas Chromatography-Mass Spectrometry) analyses were performed on a Hewlett-Packard 5973–6890 system (Palo Alto, California) operating in EI mode (70 eV) equipped with a split/splitless injector (220 °C), a split ratio 1/10, using a fused silica HP-5 MS capillary column (30 m × 0.25 mm (i.d.), film thickness: 0.25 µm) with a temperature program for HP-5 MS column from 60 °C (5 min) to 280 °C, at a rate of 4 °C/min and helium as a carrier gas at a flow rate of 1.0 mL/min. Preparative HPLC (High-Performance Liquid Chromatography) was performed using a C_18_ 25 cm × 10 mm Kromasil column on a HPLC system (Jasco PU-2080; JASCO, Tokyo, Japan) equipped with an RI detector Shimadzu 10A (Shimadzu, Kyoto, Japan); flow rate: 1.0 mL/min; concentration of the samples: 3.5–7.0 mg/mL. All solvents used were of HPLC grade (Merck). MPLC (Medium Pressure Liquid Chromatography) was performed using Büchi C-615 and Büchi 688 chromatographic pump; columns: Büchi Borosilikat 3.3, (41.0 cm × 4.0 cm), flow rate: 10 mL/min; (15.0 cm × 1.5 cm) flow rate: 3 mL/min; vacuum liquid chromatography (VLC): silica gel 60H (Merck, Art. 7736) [58]. Column chromatography (CC): silica gel (Merck, Art. 9385), silica gel 60 (230–400 mesh ASTM, SDS 2050044) gradient elution with the solvent mixtures indicated in each case; Sephadex LH-20 (Pharmacia Fine Chemicals); cellulose (Avicel, Merck, Art. 2330). Preparative TLC (Thin Layer Chromatography) was performed using pre-coated silica gel 60 plates (Merck, Art. 5721). Fractionation was always monitored by TLC silica gel 60 F-254, (Merck, Art. 5554) with visualization under UV (254 and 365 nm) and spraying with vanillin-sulfuric acid reagent (vanillin Merck, Art. No. S26047 841) [59] and Neu’s reagent for phenolics [60]. Analytical solvents were obtained from Panreac Quimica SA (Barcelone, Spain, Italy), while deuterated solvents were purchased from Merck, KGaA (Darmstadt, Germany). Di-phosphorus pentoxide was purchased from Chemlab, Belgium.

### 4.3. Extraction and Chromatography

The air-dried powdered aerial parts of *S. willeana* (0.43 kg) were successively extracted at room temperature with cyclohexane, dichloromethane, MeOH, and MeOH:H_2_O (5:1) (2 L of each solvent, twice for 48 h) [8]. A portion of the latter extract (9.0 g) was fractionated on a RP_18_-MPLC (41.0 × 4.0 cm) using a H_2_O: MeOH gradient system (100% H_2_O → 100% MeOH; steps of 10% MeOH; 50 min each; 50% MeOH: 50% EtOAc 50 min; 100% EtOAc 50 min) to yield twenty three fractions (A–V) of 500 mL each. Fraction D (598.8 mg; H_2_O:MeOH 85:15) was similarly purified by RP_18_-MPLC (15 cm × 1.5 cm) to obtain three sub-fractions (1–3). The first two sub-fractions were combined together (sub-fraction DA, 364.5 mg), subjected to a Sephadex LH-20 column and eluted with 100% methanol to afford 61 fractions combined in 13 groups (DAA–DAM). Group DAJ (4.2 mg) was identified as salvianolic acid K (**13**). Group DAC (12.9 mg) was subjected to RP_18_-HPLC (RID; isocratic elution using MeOH:CH_3_COOH 5% 30:70; flow-rate: 1.0 mL/min) and afforded 1-*O-p*-hydroxybenzoyl-*β*-d-apiofuranosyl-(1→6)-*β*-d-glucopyranoside (**8**) (Rt = 14.0 min, 0.5 mg) and 4-*O-β*-d-glucopyranosyl-acetophenone (picein) (**10**) (Rt = 17.2 min, 0.7 mg). Group DAD (9.3 mg) was purified by prep. TLC on silica gel using CHCl_3_:MeOH:AcOH (7:1.5:1.5) as the eluent and yielded hydroxytyrosol (6.3 mg) (**5**). Fraction H (718.3 mg) was fractionated by CC on a Sephadex LH-20 (25.0 cm × 3.2 cm) using H_2_O:MeOH (20:80 to 0:100) for gradient elution to afford luteolin 7-*O-β*-d-glucoside (0.7 mg) (**14**). Groups HC to HG were combined (HC′; 193.7 mg) and purified by CC (12.2 cm × 2.2 cm) over silica gel with cyclohexane: DM:EtOAc:MeOH mixtures of increasing polarity to yield nine groups (HC′A–HC′I). Group HC′C (14.2 mg; eluted with EtOAc:MeOH 97:3 to 94:6) was purified by RP_18_-HPLC (RID; MeOH; H_2_O 40:60; flow rate: 1 mL/min) to obtain syringin (Rt = 56.4 min, 0.1 mg) (**7**), (1*S*,2*R*,4*R*)-1,8-epoxy-*p*-menthan-2-yl-*O*-*β*-d-glucopyranoside (Rt = 74.2 min, 0.9 mg) (**3**), (6*R*,9*S*)-3-oxo-*α*-ionol-*β*-d-glucopyranoside (Rt = 116.5 min, 0.5 mg) (**4**), eugenyl-glucoside (Rt = 130.1 min, 0.3 mg) (**9**). Combined groups HJ to HL (HJ′; 48.9 mg; eluted with H_2_O:MeOH 50:50) were subjected to CC over silica gel using cyclohexane: DM:EtOAc:MeOH mixtures of increasing polarity (60 fractions). Purification of fraction HJ′E (2.1 mg; eluted with EtΟAc:MeOH 90:10) was carried out by prep. TLC on silica gel, using CHCl_3_:MeOH:AcOH (9.0:1.0:0.1) and afforded syringaresinol-4-*O*-*β*-d-glucopyranoside (0.9 mg) (**17**) and pinoresinol-4-*O*-*β*-d-glucopyranoside (1.2 mg) (**18**). Combined groups HM to HP (HM′; 86.7 mg) were fractionated by RP_18_-HPLC (MeOH 58%:H_2_O 42%; flow rate: 1 mL/min) and yielded rosmarinic acid (Rt = 10.6 min, 2.2 mg) (**12**) and *p*-anisic acid (Rt = 18.9 min, 0.1 mg) (**6**), and the sub-fraction HM′14b (Rt = 16.7 min, 5.9 mg), which was further purified by prep. TLC on silica gel with EtOAc:AcOH:H_2_O (65:15:20) and led to the isolation of hesperidin (3.0 mg) (**16**) and of 4-hydroxyacetophenone 4-*Ο*-(3,5-dimethoxy-4-hydroxybenzoyl)-*β*-d-glucopyranoside (2.8 mg) (**11**). Combined groups HT to HV (HT′; 176.5 mg), subjected to CC on cellulose (11.0 cm × 3.2 cm) using as eluent AcOH:H_2_O (30:70) afforded 79 fractions. Fraction HT′H (4.8 mg) was purified by prep. TLC on silica gel with EtOAc:AcOH:H_2_O (65:15:20) to obtain 6-hydroxyluteolin 7-*O*-*β*-d-glucoside (0.9 mg) (**15**). The purification of fraction N (248.5 mg) was performed on silica gel CC (15.2 cm × 2.0 cm) using mixtures of cyclohexane: DM:EtOAc:MeOH of increasing polarity and afforded methyl *α*-linolenate (C18:3) (1.4 mg) (**20**) and ursolic acid (4.7 mg) (**1**). Groups NB′ (7.4 mg) and NF (19.6 mg) were subjected to prep. TLC on silica gel with CHCl_3_:MeOH:AcOH (9.5:0.5:0.05) and yielded linoleic acid (C18:2) (0.5 mg) (**19**) and maslinic acid (18.3 mg) (**2**), respectively. All obtained extracts, fractions, and isolated compounds were evaporated to dryness in vacuum under low temperature and then were put in activated desiccators with P_2_O_5_ until their weights had stabilized.

### 4.4. NMR Data of ***5***, ***8***, and ***13***

*Compound* **5**: Yellow amorphous powder; ^1^H-NMR (CD_3_OD, 400 MHz): 2.66 (2H, t, *J =* 7.3, H-7), 3.66 (2H, t, *J =* 7.3, H-8), 6.52 (1H, dd, *J =* 8.0, 2.0, H-6), 6.65 (1H, d, *J =* 2.0, H-2), 6.67 (1H, d, *J =* 8.0, H-5).

*Compound* **6**: White amorphous powder; ^1^H-NMR (CD_3_OD, 400 MHz): 3.91 (3H, s, OCH_3_), 6.85 (2H, d, *J =* 8.5, H-3/H-5), 7.89 (2H, d, *J =* 8.5, H-2/H-6).

*Compound* **8**: White amorphous powder; ^1^H-NMR (CD_3_OD, 400 MHz): *δ* 3.38 (1H, m, H-5′), 3.47 (2H, m, H-2′, H-3′), 3.56 (2H, s, H-5a,b″), 3.61 (1H, m, H-6b′), 3.73 (1H, d, *J* = 9.6, H-4b″), 3.90 (1H, d, *J* = 2.2, H-2″), 3.96 (1H, d, *J* = 9.6, H-4a″), 3.99 (1H, d, *J* = 11.5, H-6a′), 4.96 (1H, d, *J* = 2.2, H-1″), 5.64 (1H, d, *J* = 7.9, H-1′), 6.84 (2H, d, *J* = 8.7, H-3, H-5), 7.96 (2H, d, *J* = 8.7, H-2, H-6).

*Compound* **13**: Yellow amorphous powder; ${\left[a\right]}_{D}^{20}$ + 0.36° (c 0.350 MeOH); ^1^H-NMR (CD_3_OD, 400 MHz): *δ* 2.90 (1H, dd, *J* = 14.0, 9.8, H-7′), 3.07 (1H, dd, *J =* 14.0, 3.8, H-7′), 4.24 (1H, d, *J =* 6.5, H-8″), 4.88 (1H, d, *J =* 6.5, H-7″), 5.02 (1H, dd, *J =* 9.8, 3.8, H-8′), 6.31 (1H, d, *J =* 16.0, H-8), 6.38 (1H, d, *J =* 8.0, H-5), 6.62 (1H, dd, *J =* 8.0, 1.6, H-6′), 6.66 (1H, d, *J =* 8.0, H-5′), 6.74 (1H, d, *J =* 8.0, H-5″), 6.76 (1H, s, H-2′), 6.82 (1H, dd, *J =* 8.0, 2.0, H-6), 6.84 (1H, dd, *J =* 8.0, 2.0, H-6″), 6.98 (1H, d, *J =* 2.0, H-2″), 7.01 (1H, d, *J =* 2.0, H-2), 7.47 (1H, d, *J =* 16.0, H-7). 

## Figures and Tables

**Table 1:** ^1^H-NMR and ^13^C-NMR spectrum of **11**.

	*δ*_C_	C	*δ*_H_	H	*J* (Hz)
1	131.5	C	-	-	-
2	130.1	CH	7.69	1	d (*J* = 9.0)
3	116.2	CH	7.05	1	d (*J* = 9.0)
4	162.5	C	-	-	-
5	116.2	CH	7.05	1	d (*J* = 9.0)
6	130.1	CH	7.69	1	d (*J* = 9.0)
7	199.1	C	-	-	-
8	26.5	CH_3_	2.47	3	s
1′	101.7	CH	5.04	1	d (*J* = 7.8)
2′	74.5	CH	3.53	2	m
3′	77.5	CH			
4′	71.3	CH	3.43	1	m
5′	75.5	CH	3.89	1	dd (*J* = 8.0, 2.3)
6a′	64.8	CH_2_	4.71	2	dd (*J* = 11.7, 2.3)
6b′			4.46		dd (*J* = 11.7, 8.0)
1″	-	C	-	-	-
2″	108.3	CH	7.33	1	s
3″	149.0	C	-	-	-
4″	142.3	C	-	-	-
5″	149.0	C	-	-	-
6″	108.3	CH	7.33	1	s
7″	167.3	C	-	-	-
3″, 5″-OCH_3_	56.0	CH_3_	3.83	6	s

## Supplementary Materials

The following are available online at http://www.mdpi.com/2223-7747/7/1/18/s1. Table S1: Non-volatile secondary metabolites of *Salvia* L.
