# Supplementary material for: Polar Constituents of Salvia willeana (Holmboe) Hedge, Growing Wild in Cyprus
Source: Plants (Basel). 2018 Mar 6;7(1):18. doi: 10.3390/plants7010018 (PMC5874607; doi:10.3390/plants7010018)
Supplement: Supplementary file 1 [file plants-07-00018-s001.pdf]

SUPPLEMENTARY MATERIALS

Polar constituents of *Salvia willeana* (Holmboe) Hedge, growing wild in Cyprus.

Mailis Theofilos[1], SkaltsaHelen[1,*]

[1]Department of Pharmacognosy & Chemistry of Natural Products, Faculty of Pharmacy, University of Athens, Panepistimiopolis, Zografou, Athens, 157 71 Greece.

* Corresponding author. Tel., fax: +30 210 7274593.

E-mail address: skaltsa@pharm.uoa.gr

Table of Contents **Page**

S1: ^{1}H-NMR (400 MHz, CD_3OD) Spectrum of Compound **8** 3
S2: COSY-90 (400 MHz, CD_3OD) Spectrum of Compound **8** 4
S3: ^{1}H-NMR (400 MHz, CD_3OD) Spectrum of Compound **11** 5
S4: COSY-90 (400 MHz, CD_3OD) Spectrum of Compound **11** 6
S5: HSQC (400 MHz, CD_3OD) Spectrum of Compound **11** 7
S6: HMBC (400 MHz, CD_3OD) Spectrum of Compound **11** 8
S7: ROESY (400 MHz, CD_3OD) Spectrum of Compound **11** 9
S8: ^{1}H-NMR (400 MHz, CD_3OD) Spectrum of Compound **13** 10
S9: HSQC (400 MHz, CD_3OD) Spectrum of Compound **13** 11
S10: HMBC (400 MHz, CD_3OD) Spectrum of Compound **13** 12
S11: COSY (400 MHz, CD_3OD) Spectrum of Compound **13** 13
S12: COSY & HMBC signals of Compound **13** 14
S13: Table S1. Non volatile secondary metabolites of *Salvia* L. 15

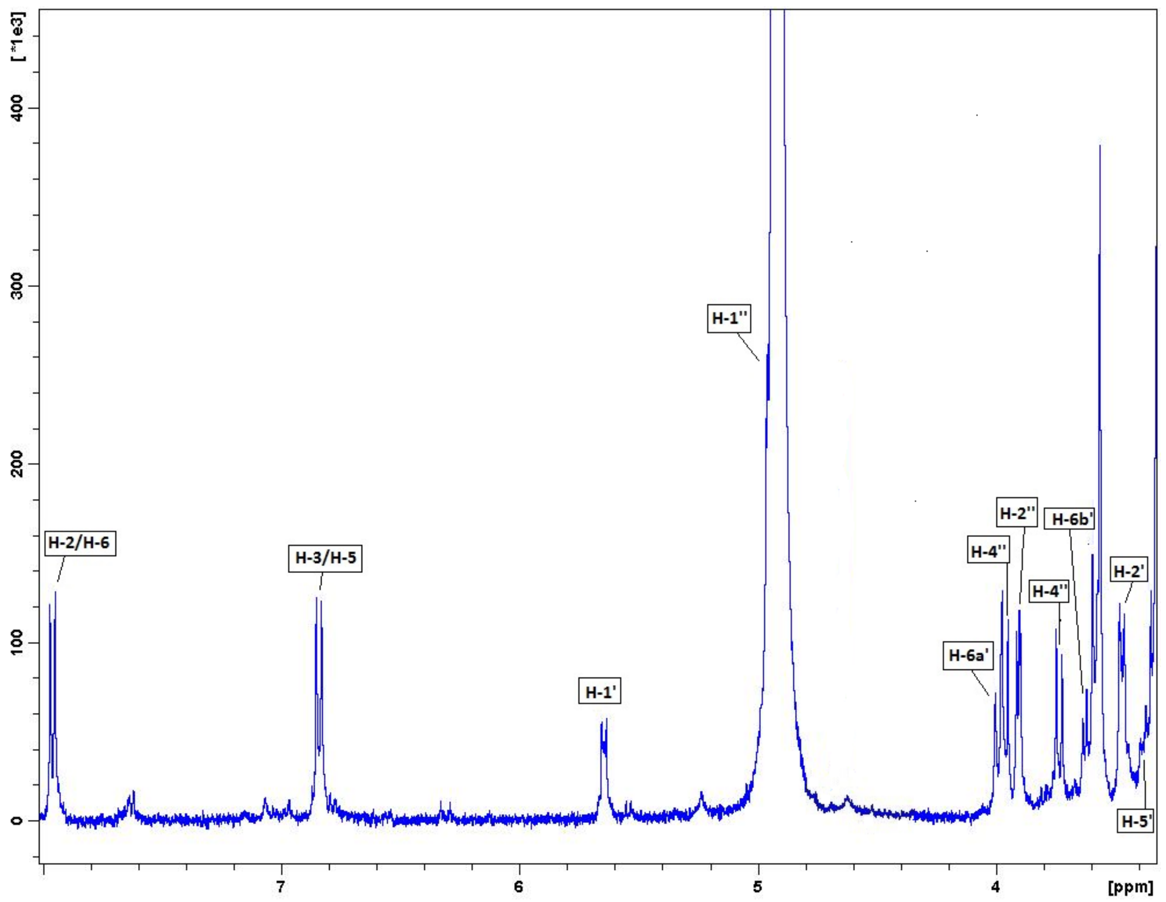

^{1}H –NMR spectrum of **8** (CD_3OD, 400 Hz)

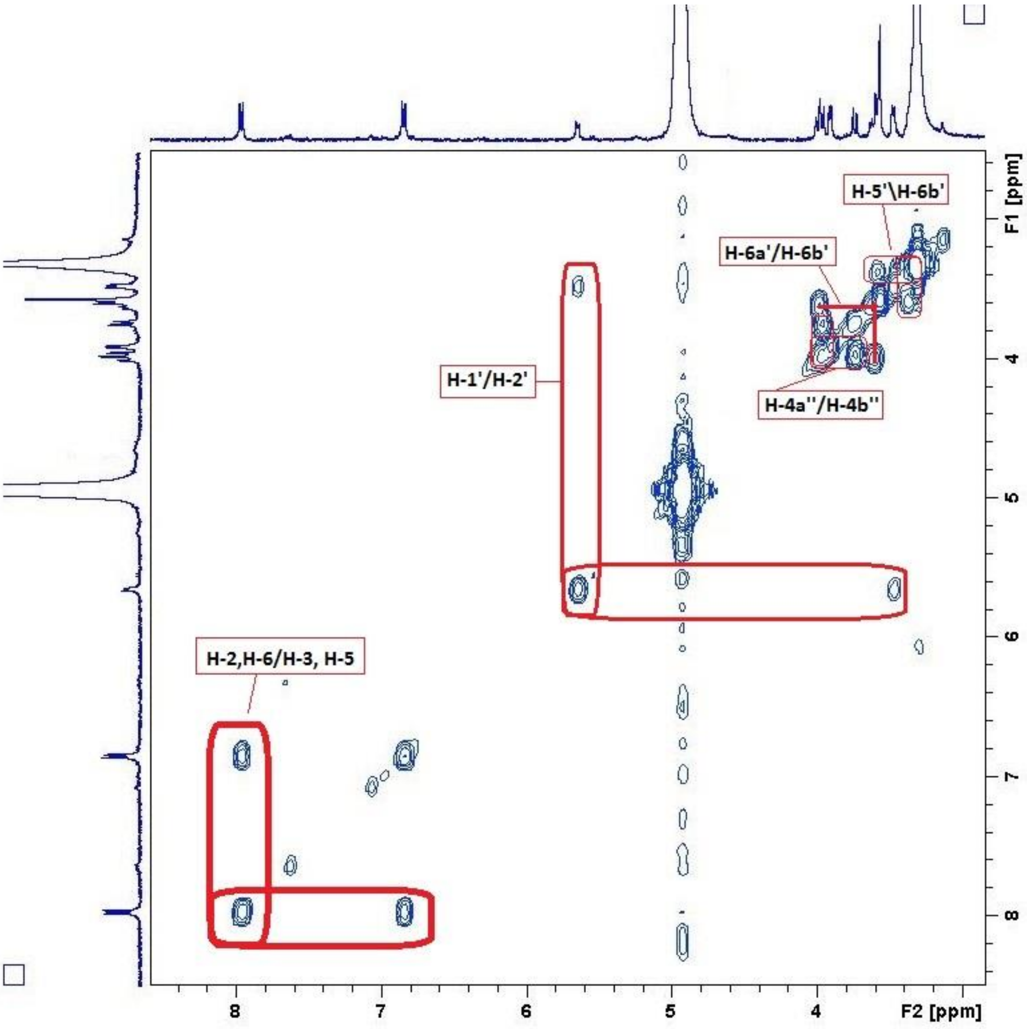

COSY spectrum of **8** (CD_3OD, 400 Hz)

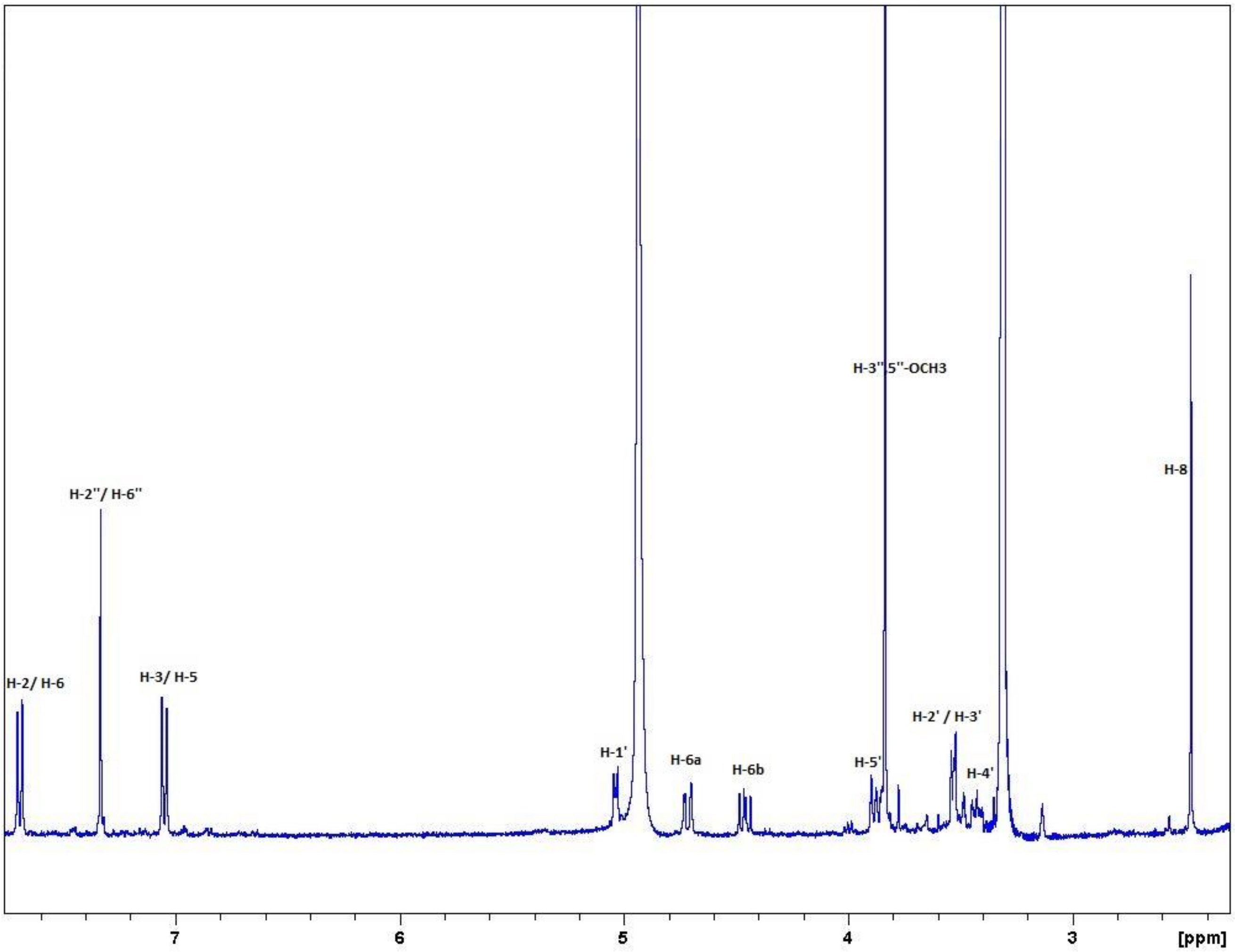

^{1}H –NMR spectrum of **11** (CD_3OD, 400 Hz)

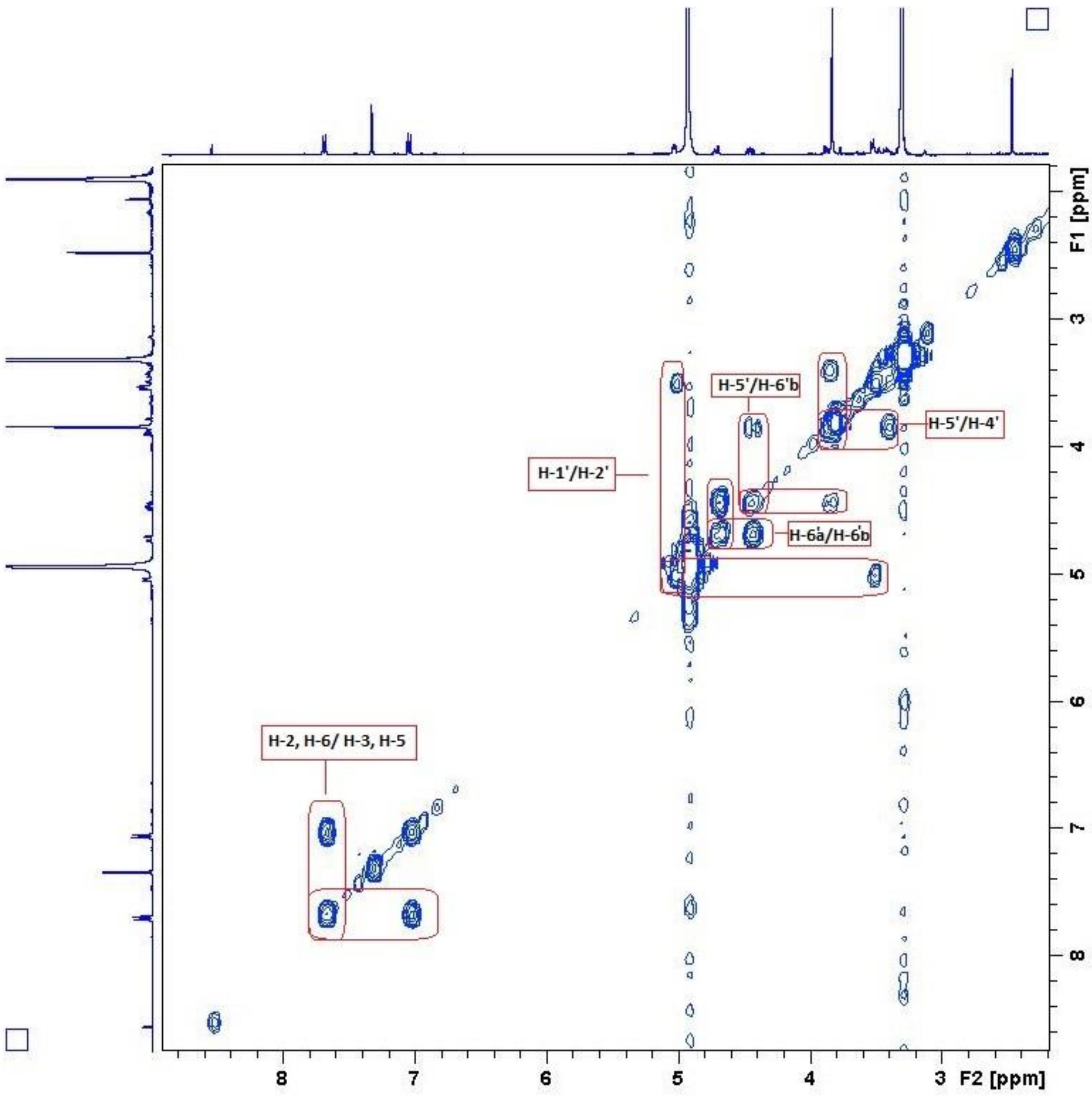

COSY spectrum of **11** (CD_3OD, 400 Hz)

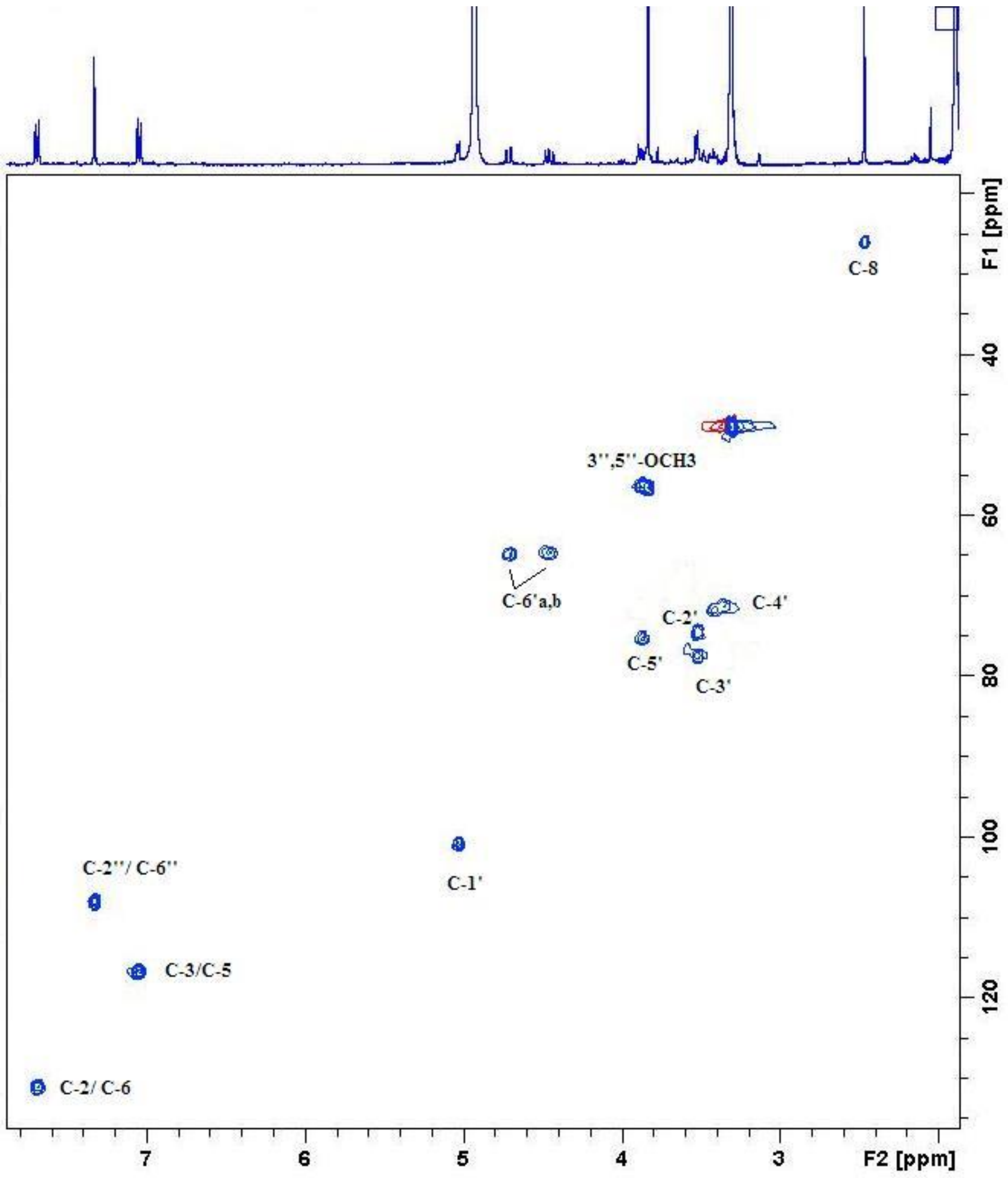

HSQC spectrum of **11** (CD_3OD, 400 Hz)

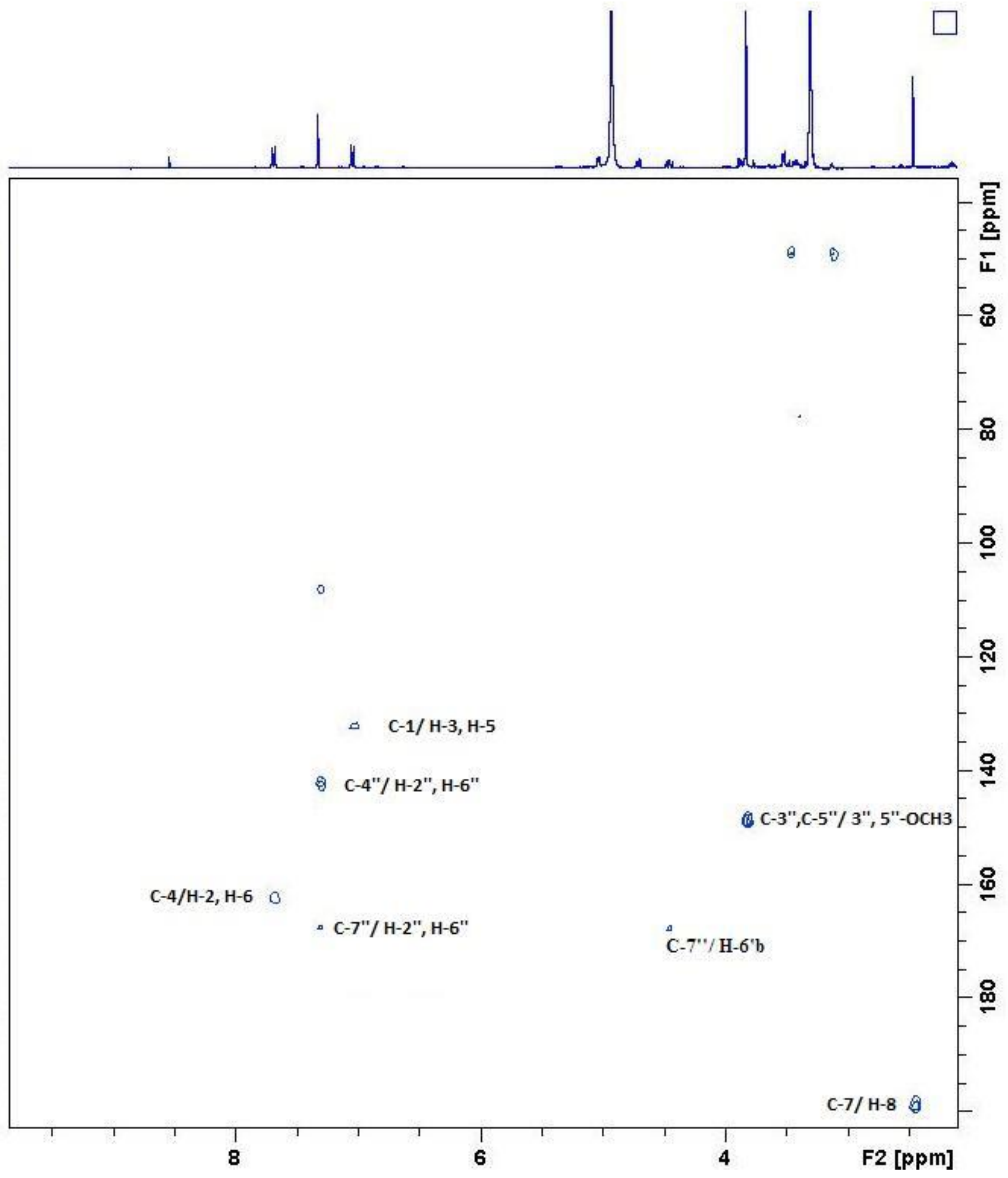

HMBC spectrum of **11** (CD_3OD, 400 Hz)

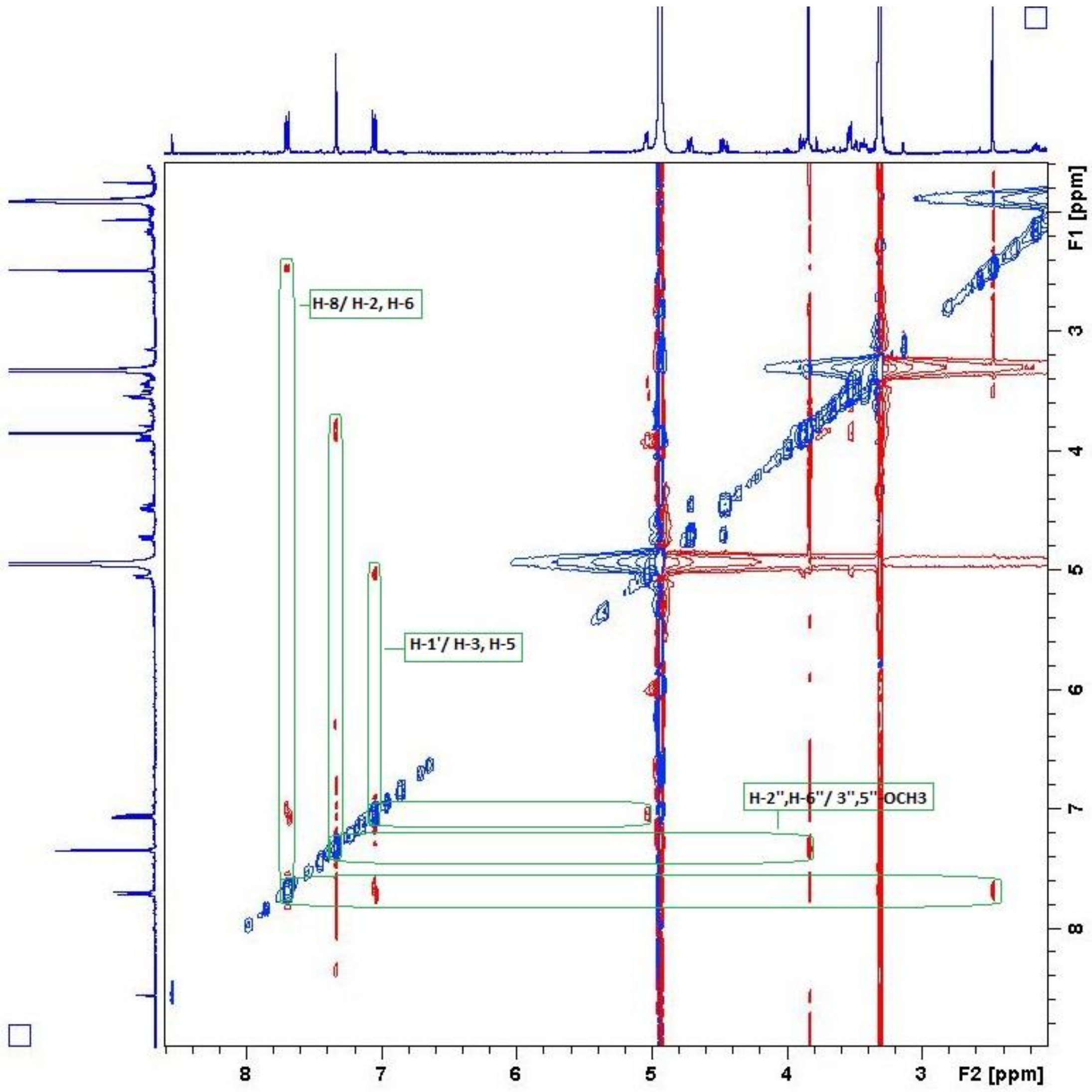

ROESY spectrum of **11** (CD_3OD, 400 Hz)

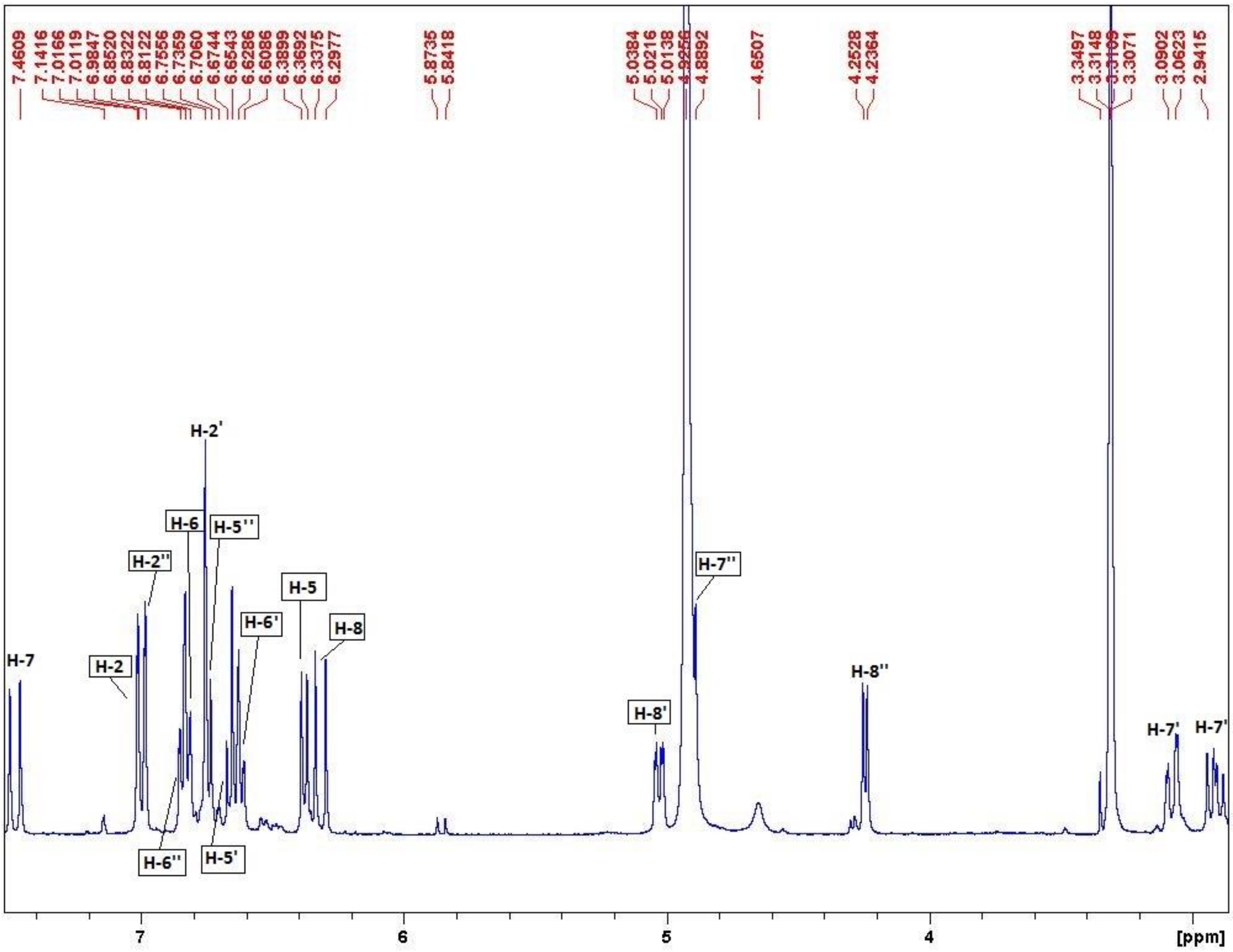

^{1}H –NMR spectrum of **13** (CD_3OD, 400 Hz)

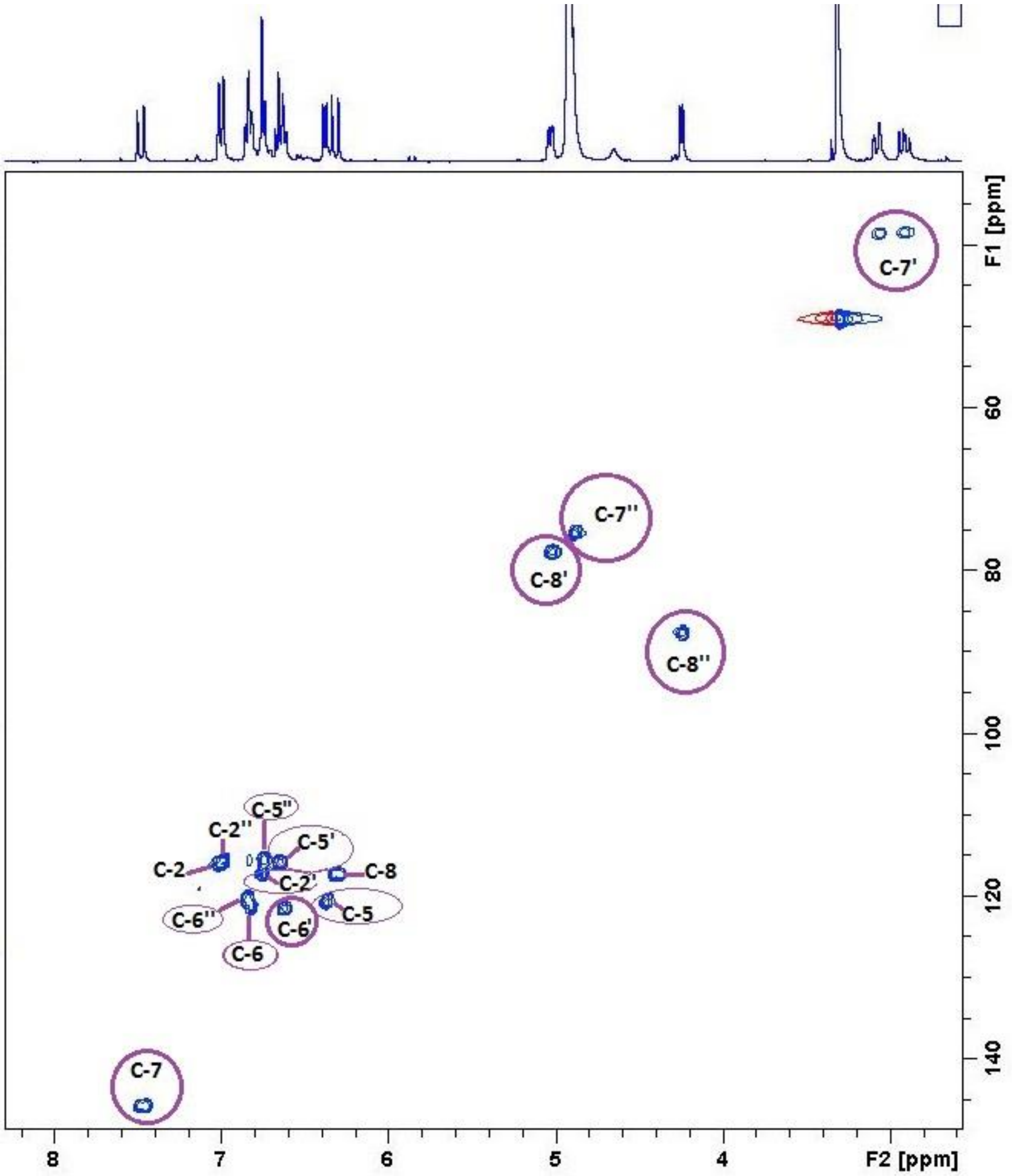

HSQC spectrum of **13** (CD_3OD, 400 Hz)

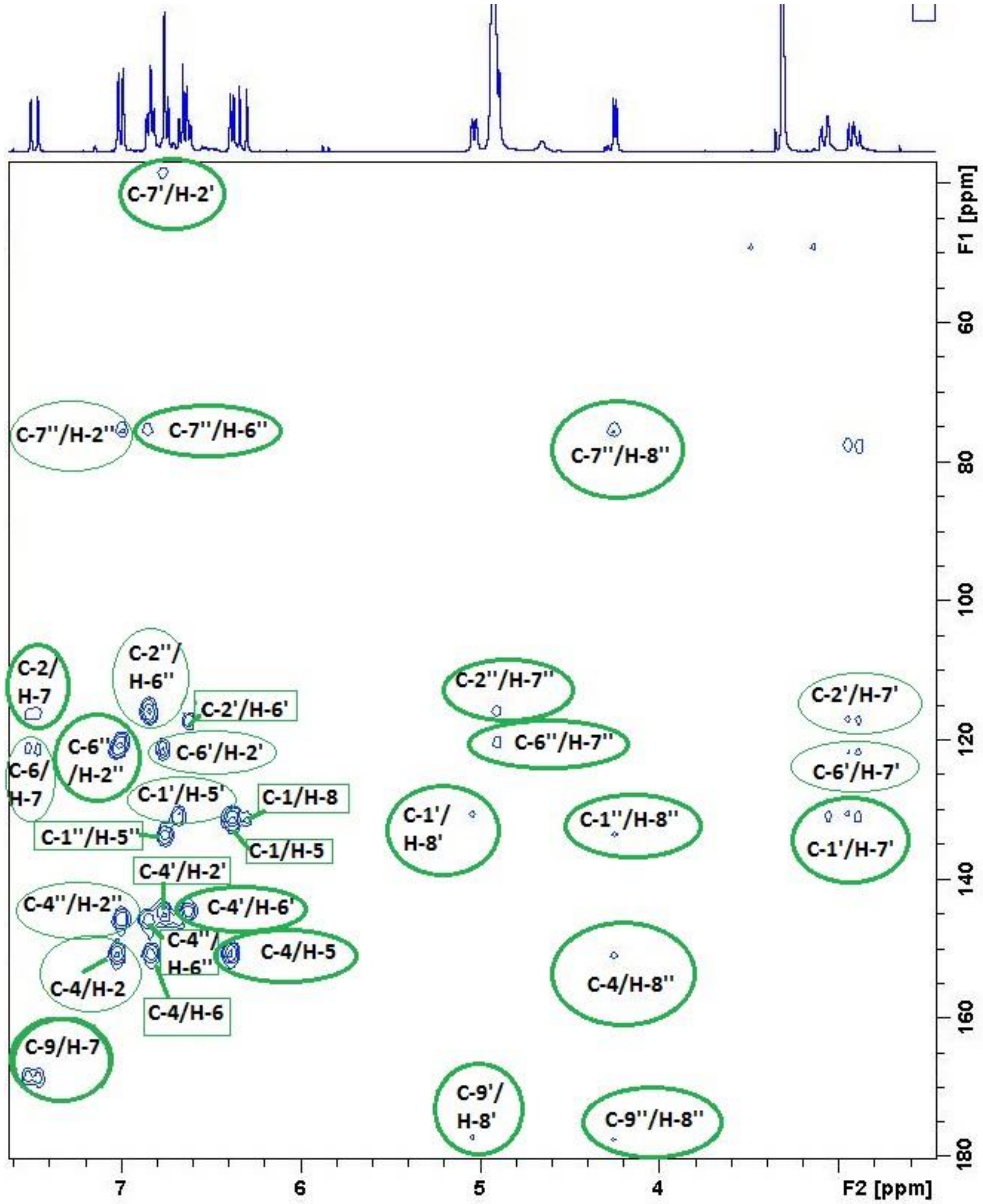

HMBC spectrum of **13** (CD_3OD, 400 Hz)

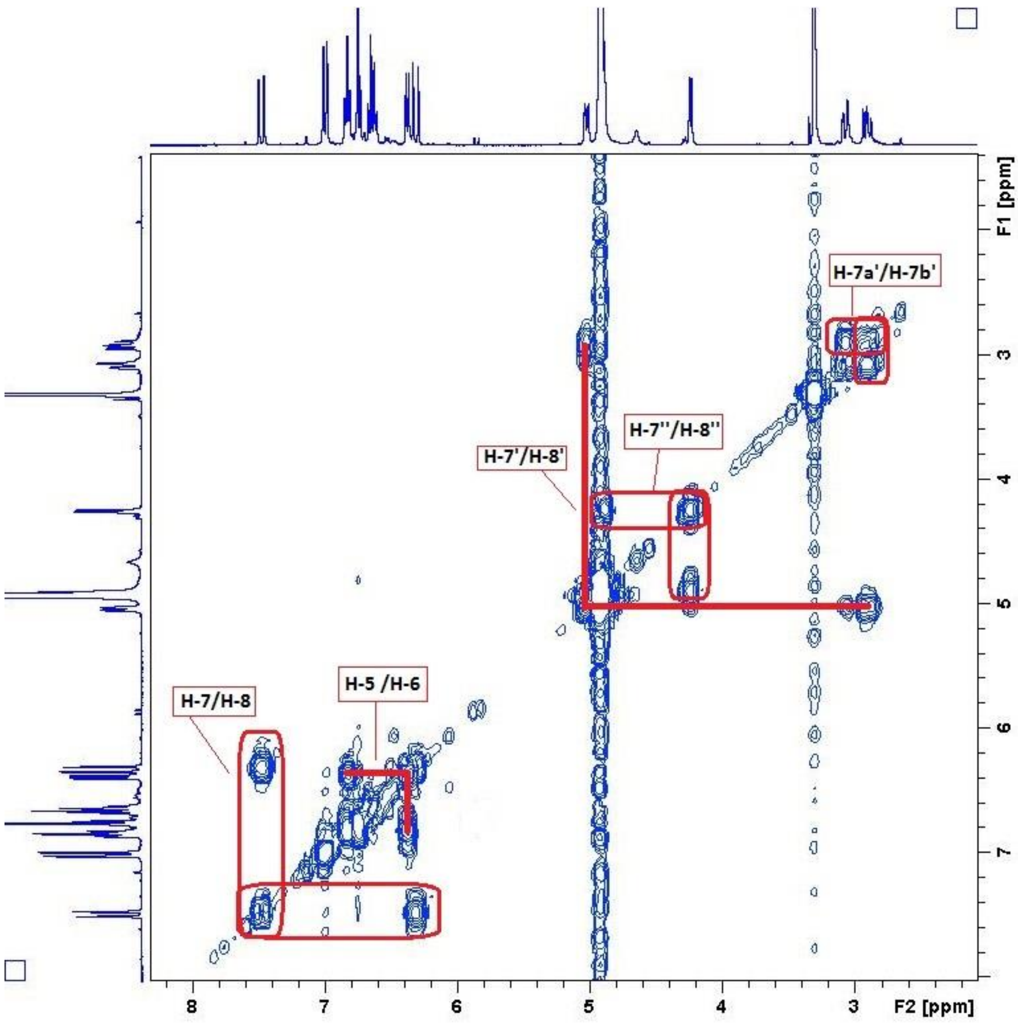

COSY spectrum of **13** (CD_3OD, 400 Hz)

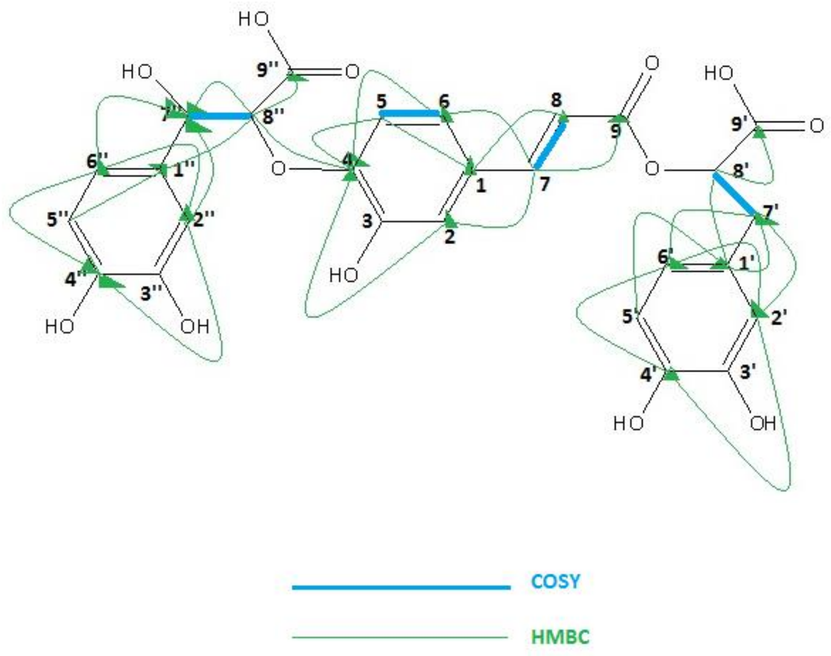

COSY & HMBC signals of compound **13**

Table S1. Non volatile secondary metabolites of *Salvia* L.

**S1.1. Phenols and hydroxycinnamic acids**		
S. candidissima Vahl	2,4-dimethoxy-benzoic acid	[1]
S. cavaleriei H.Lév.	salvianolic acids A, C, H, I<br>isosalvianolic acid C<br>rosmarinic acid<br>lithospermic acid	[2, 3]
S. digitaloides Diels	hydroxy-tyrosol	[4]
S. flava Forrest ex Diels	salviaflaside	[5]
S. flava Forrest ex Diels	rosmarinic acid, salvianolic acid J	[6]
S. miltiorrhiza Bunge	isoferulicacid<br>3,4-dihydroxyphenyllacticacid = danshensu<br>3,4-dihydroxy-phenyl-lactamide<br>rosmarinic acid	[7-10]
S. miltiorrhiza Bunge	salvianolic acids A-G	[7, 11-14]
S. officinalis L.	sagerinic acid, 6-feruloyl-α-glucose<br>6-caffeoyl-1-fructosyl-α-glucoside<br>1-*p*-hydroxybenzoyl-6-apiosylglucoside<br>2-(3-mehtoxy-4-glucosyloxyphenyl)-3-hydroxymethyl-5-(3-hydroxypropyl)-7-methoxy-2,3-dihydrobenzofuran<br>syringic acid, astringin<br>*trans*-resveratrol, *cis*-resveratrol	[15-18]
S. officinalis L.	salvianolic acids K, L, Y	[15, 19, 20]
S. officinalis L.	caffeic acid, ferulic acid, 4-hydroxybenzoic acid, vanillic acid, rosmarinic acid	[15, 21, 22]
S. officinalis L.	4-methoxybenzoic acid (*p*-anisic acid)	[23]
S. prionitis Hance	prionitisides A, B, rosmarinic acid	[24]
S. przewalskii Maxim.	przewalskinic acid A<br>dimethyl lithospermate B	[25]<br>[26]
S. sclareoides Brot.	caffeic acid, ferulic acid, vanillic acid, gentisic acid, chlorogenic acid	[27]
S. sonchifolia C.Y.Wu	protocatechuic acid, caffeic acid<br>3,4-dihydroxyphenyllacticacid = danshensu<br>rosmarinic acid, lithospermic acid	[28]
S. yunnanensis C.H.Wright	yunnaneic acids A-E	[29, 30]
**S1.2. Coumarins and acetophenones**		
S. aegyptiaca L.	7-methoxycoumarin (herniarin)	[31]
S. cedronella Boiss.	3-methoxy-4-hydroxy-7-methyl-coumarin	[32]
S. euphratica Montbret & Aucher ex Benth.	6,7-dihydroxycoumarin (esculetin)	[33]
S. officinalis L.	sagecoumarin	[34]

S. officinalis L.	4-hydroxyacetophenone-4-glucoside (picein)<br>4-hydroxyacetophenone-4-(6-apiosyl)glucoside<br>4-hydroxyacetophenone-4-(2-(5-syringoyl)apiosyl) glucoside	[16, 21, 35]
S.plebeia R.Br.	scopoletin	[36]
**S1.3. Lignans**		
S. chinensis Benth.	syringaresinol	[36]
S. officinalis L.	1-hydroxypinoresinol-1-*O*-glucoside<br>isolariciresinol-3*α*-glucoside	[37]
S. plebeia R.Br.	feruloylisolariciresinol 12-methylmyristate<br>isolariciresinol di (12-methylmyristate)	[38, 39]
S. santolinifolia Boiss.	salvicins A, B<br>santolinol, didemethylpinoresinol	[40,41]
S. scapiformis Hance	(+)-medioresinol, (+)-pinoresinol<br>(+)-8*α*-hydroxypinoresinol<br>(+)-8*α*-hydroxypinoresinol-8-*O*-*β*-D-glucoside<br>(+)-8*α*-hydroxypinoresinol-8-*O*-*β*-D-[6′′-*O*-(4′′′-hydroxybenzoyl)]-*β*-D-glucoside	[42]
**S1.4. Flavonoids** [43]		
**S1.4.1.Flavones**		
S. blepharophylla Brandegee ex Epling	6-hydroxyluteolin-7-methyl ether (pedalitin) 6-hydroxyluteolin-7-4'-dimethyl ether (nuchensin)	[44]
S. candidissima Vahl.	chrysoeriol<br>luteolin-4'-methyl ether (diosmetin)	[45]
S. cardiophylla Benth.	6-hydroxyluteolin-6,3',4'-trimethylether (eupatilin)<br>6-hydroxyluteolin-6,7,3',4'-tetramethylether	[46]
S. hypoleuca Benth.	apigenin-7, 4'-dimethyl ether<br>luteolin<br>6-hydroxyapigenin-6,4'-dimethyl ether (pectolinarigenin)<br>6-hydroxyapigenin-7,4'-dimethyl ether<br>6-hydroxyapigenin-6,7,4'-trimethyl ether (salvigenin)<br>6-hydroxyluteolin-6,7-dimethyl ether (cirsiliol)	[47]
S. lavandulaefolia Vahl	apigenin, genkwanin,<br>5,7,3',4'-tetrahydroxyflavone (luteolin)<br>luteolin-7-methylether<br>luteolin-3'-methyl ether (chrysoeriol)<br>6-hydroxyluteolin-6,7,3'-trimethyl ether (cirsilineol)<br>6-hydroxyluteolin-6,7,4'-trimethyl ether (eupatorin)	[48]
S. nicolsoniana Ramamoorthy	genkwanin, acacetin, apigenin-7, 4'-dimethyl ether<br>luteolin-3',4'-dimethyl ether	[49]
S. officinalis L.	apigenin, genkwanin, apigenin-7, 4'-dimethyl ether<br>luteolin, luteolin-7-methyl ether<br>6-hydroxyapigenin (scutellarein)<br>6-hydroxyapigenin-6-methyl ether (hispidulin)<br>6-hydroxyapigenin-6,7-dimethyl ether (cirsimaritin)	[22, 50]

S. officinalis L.	6-hydroxyapigenin-5,6,7,4'-tetramethyl ether	[51]
S. officinalis L.	6-hydroxyluteolin-6-methyl ether (nepetin, eupafolin)	[50]
S. officinalis L.	8-hydroxyapigenin (isoscutellarein)	[22]
S. palaestina Benth.	apigenin, genkwanin, apigenin-7, 4'-dimethyl ether<br>luteolin, chrysoeriol, luteolin-7,4'-dimethyl ether<br>6,7,3',4'-tetramethoxyflavone	[52]
S. plebeian R.Br.	hispidulin, nepetin,eupafolin, eupatorin<br>8-hydroxyapigenin-7-methyl ether (salvitin)	[53-55]
S. tomentosa Mill.	5-hydroxy-6,7,3',4'-tetramethoxyflavone, cirsilineol, jaceosidin, 6-methoxy-luteolin	[92]
S. triloba L.f.	salvigenin, 6-hydroxyluteolin-6,3'-dimethyl ether (jaceosidin)	[56, 57]
S. virgata Jacq.	luteolin-7,3',4'-trimethyl ether	[58]
S. willeana (Holmboe) Hedge	6-hydroxyapigenin-6,7,4'-trimethyl ether (salvigenin)	[59]
S. yosgadensis Freyn & Bornm.	5,7,4'-trihydroxyflavone(apigenin)<br>apigenin-7-methyl ether (genkwanin)<br>apigenin-4'-methyl ether (acacetin)<br>apigenin-7, 4'-dimethyl ether	[1]
	**S1.4.2. Dihydroflavones**	
S. miltiorrhiza Bunge	5,3'-dihydroxy-7,4'-dimethoxyflavanone	[60]
S. nicolsoniana Ramamoorthy	5,7-dihydroxy-4'-methoxyflavanone (isosakuranetin)	[49]
*S. officinalis*L.	5,7,3'-trihydroxy-4'-methoxyflavanone (hesperitin)	[22]
S. texana (Scheele) Torr.	5-hydroxy-7-methoxyflavanone	[61]
	**S1.4.3. Flavonols**	
S. columbariae Benth.	6-hydroxy-kaempferol-5,6-dimethyl ether<br>6-hydroxygalangin-5,6-dimethyl ether	[47]
S. compressa Vent.	quercetin-3-methyl ether	[47]
S. cyanescens Boiss. & Balansa	kumatakenin<br>6-hydroxykaempferol-3,6-dimethyl ether	[62]
S. dorrii (Kellogg) Abrams	5,7,4'-trihydroxyflavonol (kaempferol)<br>5,7,3',4'-tetrahydroxyflavonol (quercetin)	[47]
S. farinacea Benth.	quercetin-3'-methyl ether (isorhamnetin)	[67]
S. glutinosa L.	kaempferol-3-methyl ether (isokaempferide)<br>kaempferol-3,7-dimethyl ether (kumatakenin)<br>quercetin-3,7,4'-trimethyl ether (ayanin)<br>quercetin-3,7,3',4'-tetramethyl ether (retusin)	[63]
S. longipedicellata Hedge	6-hydroxykaempferol-3,6,4'-trimethyl ether (santin)<br>quercetin-3,3'-dimethyl ether	[33]
	**S1.4.4. Glycosides**	
S. aegypteacae L.	apigenin-7-glucoside (cosmosiin),<br>luteolin-7-glucoside (cinaroside)	[64]

	luteolin-7-cellobioside, luteolin-6,8-di-C-glucoside	
S. blepharophylla Brandegee ex Epling	apigenin-8-C-glucoside (vitexin)<br>apigenin-8-C-arabinoside (schaftoside)<br>quercetin-3-glucoside (isoquercetin)<br>quercetin-3-glucuronide (miquelianin)<br>quercetin-7-methyl ether-3-glucoside (rhamnetin-3-glucoside), quercetin-3-robinoside	[44]
S. cavalerei H.Lév.	kaempferol-3-glucoside (astragalin)	[65]
S. coccinea Bucˈhoz ex Etl.	cyanidin-3-(6-caffeoylglucoside)-5-(4,6-dimalonylglucoside)<br>cyanidin-3-(6-caffeoylglucoside)-5-(6-malonyl-glucoside)<br>cyanidin-3-(6-caffeoylglucoside)-5-glucoside<br>cyanidin-3-(6-p-coumaroyl-glucoside)-5-(4,6-dimalonylglucoside)<br>cyanidin-3-(6-p-coumaroyl-glucoside)-5-(6-malonyl-glucoside)<br>cyanidin-3-(6-p-coumaroyl-glucoside)-5-glucoside	[66]
S. farinacea Benth.	kaempferol-3-(2^{G}-rhamnosylrutinoside)<br>kaempferol-3-robinoside<br>quercetin-3ˈ-methyl ether-3-galactosidequercetin-3ˈ-methylether-3-(2^{G}-rhamnosylrutinoside)	[67]
S. farinacea Benth.	malvidin-3-(6-p-coumaroyl-glucoside)-5-(6-malonyl-glucoside) (salviamalvin)	[68]
S. horminum L.	apigenin-7-glucoside, apigenin-7-rutinoside	[69]
S. lavandulifolia Vahl	luteolin-7-glucoside, luteolin-4ˈ-glucuronide, luteolin-7-rutinoside	[70]
S. lavandulifolia ssp. *oxyodon* = *S. officinalis* ssp. *oxyodon* (Webb & Heldr.) Reales, D. Rivera & Obón	luteolin-5-rutinoside	[71]
S. officinalis L.	apigenin-7-glucoside, luteolin-7-glucoside, luteolin-7-glucuronide, luteolin-3ˈ-glucuronide<br>6-hydroxy-luteolin-7-glucoside<br>6-hydroxy-luteolin-7-glucuronide<br>apigenin-6,8-δι-C-glucoside (vicenin-2)<br>salvigenin	[35, 72, 81]
S. palaestina Benth.	apigenin-7-glucoside, luteolin-7-glucoside, luteolin-7-glucuronide<br>luteolin-3ˈ-methyl ether-7-glucoside (chrysoeriol-7-glucoside), luteolin-3ˈmethyl ether-7-glucuronide	[52]
S. patens Cav.	apigenin-7,4ˈ-diglucoside	[73]
Salvia spp.	apigenin-7-xyloside	[74]
S. splendens Sellow ex Schult.	pelargonidin-3-(6-caffeoylglucoside)-5-(4,6-dimalonylglucoside) (salvianin)<br>pelargonidin-3-(6-caffeoylglucoside)-5-(6-malonyl-glucoside)<br>pelargonidin-3-(6-caffeoylglucoside)-5-glucoside	[68, 75]

	pelargonidin-3-(6-p-coumaroyl-glucoside)-5-(4,6-dimalonylglucoside) (monardaein)<br>pelargonidin-3-(6-p-coumaroyl-glucoside)-5-(6-malonyl-glucoside)<br>pelargonidin-3-(6-p-coumaroyl-glucoside)-5-glucoside	
S. splendens Sellow ex Schult.	delphinidin-3-(6-caffeoylglucoside)-5-(4,6-dimalonylglucoside) (salviadelphin)<br>delphinidin-3-(6-caffeoylglucoside)-5-(6-malonyl-glucoside)<br>delphinidin-3-(6-caffeoylglucoside)-5-glucoside<br>delphinidin-3-(6-p-coumaroyl-glucoside)-5-(4,6-dimalonylglucoside)<br>delphinidin-3-(6-p-coumaroyl-glucoside)-5-glucoside (awobanin)	[68, 76]
S. triloba L.f.	apigenin-7-glucoside, apigenin-7-glucuronide, luteolin-7-glucoside, luteolin-3'methylether-7-glucuronide, luteolin-3'-glucoside-7-glucuronide<br>luteolin-7-cellobioside,<br>6-hydroxy-apigenin-6-methylether-7-glucoside<br>6-hydroxy-apigenin-6-methylether-7-glucuronide<br>6-hydroxy-luteolin-6-methyl ether-7-glucoside (nepitrin)<br>6-hydroxy-luteolin-6-methyl ether-7-glucuronide	[57]
S. uliginosa Benth.	apigenin-7,4'-diglucoside, apigenin-7-cellobioside<br>apigenin-7- cellobioside-4'-glucoside	[77]
S. uliginosa Benth.	delphinidin-3-(6-p-coumaroyl-glucoside)-5-(6-malonyl-glucoside)<br>delphinidin-3-(6-p-coumaroyl-glucoside)-5-(4-acetyl-6-malonyl-glucoside)	[78]
S. verbenaca L.	luteolin-7-glucoside<br>6-hydroxy-luteolin-6,7-dimethyl ether-5-glucoside	[64, 79]
S. verticillata L.	luteolin-7-glucoside, 6-hydroxy-luteolin-5-glucoside, 6-hydroxy-apigenin-6,7,4'-trimethylether-5-glucoside (salvigenin-5-glucoside)	[80]
**S1.5. Anthaquinones**		
S. digitaloides Diels	chysophanol, physcion, emodin	[4]
S. officinalis L.	physcion	[81]
*S. przewalskii*Maxim.	Przewalskinone B, ziganein	[82]
**S1.6. Terpenes**		
**S1.6.1. Iridoids, SLs**		
S. digitaloides Diels	salvialosides A-E	[4]
S. plebeian R.Br.	1α-acetoxy-8α-hydroxy-2-oxoeudesman-3,7(11)-dien-8,12-olide<br>1α-acetoxy-8α,9β-dihydroxy-2-oxo-eudesman-3,7(11)-dien-8,12-olide	[83]

S1.6.2. Diterpenes and sesterterpenes		
S. albocaerulea Lindl.	15-hydroxy-7-oxo-abieta, 8,11,13-triene, sugiol	[84]
S. ballotaeflorae Benth.	conacytone	[85]
S. digitaloides Diels	salviatalin A, salvitrijudin A	[86]
S. divinorum Epling & Játiva	Salvinorins A-F, divinatorins A-C	[87, 88]
S. dorrii (Kellogg) Abrams	salvidorol	[89]
S. forskahlei L.	forskalinone	[90]
S. heldrichiana Boiss.	isopimaric acid, 7β-hydroxysandaracopimaric acid<br>7-oxo-13-epi-pimara-8,15-dien-18-oic acid<br>wiedelactone, wiedemannic acid, heldrichinic acid	[91]
S. herbacea Benth.	tehuanins A-I, acetyltehuanin C,<br>1(10)-dehydrosalviarin, 1α,10α-epoxysalviarin,<br>16-bromotehuanin F	[92]
S. hypargeia Fisch. & C.A.Mey.	hypargenins A-F	[93]
S. miltiorrhiza Bunge	tanshinones I, II<br>cryptotanshinone, danshexinkun A<br>1,2-dihydrotanshinquinone	[94]
S. nemorosa L.	nemorone	[84]
S. officinalis L.	sagequinone methide A, carnosol, manool,<br>6,7-dimethoxy-7-epi-rosmanol, 7-methoxy-rosmanol	[95]
S. officinalis L.	rosmadial, epi-rosmanol	[96]
S. officinalis L.	columbaridione, 12-*O*-methylcarnosic acid,<br>galdosol, atuntzensin A, miltirone, safficinolide<br>sageone, dimethyl sageone<br><br>rel-(5*S*, 6*S*, 7*S*, 10*R*, 12*S*, 13*R*)-7-hydroxyapiana-8,14-diene-11,16-dion-(22,6)-olide<br>rel-(5*S*, 6*S*, 7*R*,10*R*, 12*S*, 13*R*)-7-hydroxyapiana-8,14-diene-11,16-dion-(22,6)-olide<br>rel-(5*S*, 6*S*, 7*S*, 10*R*, 12*R*, 13*S*)-7-hydroxyapiana-8,14-diene-11,16-dion-(22,6)-olide<br>carnosol, rosmadial, rosmanol, epirosmanol, isorosmanol, columbaridione, atuntzensin A, miltirone, carnosic acid, 12-*O*-methyl carnosic acid	[95]<br><br><br><br>[81]
S. palaestina Benth.	2-oxocandesalvone A<br>12-*O*-methyl-2-oxocandesalcone A<br>12-*O*-methyl-candesalvone A<br>methyl 12-*O*-methyl-saluipalestinoate<br>salvipalestinoic acid, candelabrone	[97]
S. plebeia R.Br.	carnosic acid, methyl carnosate, rosmanol	[98]
S. przewalskii Maxim.	salprzelactone, dehydrodanshenol A,<br>deacetylsalvianonol, isograndifoliol	[99]
S. regla Cav.	sessein, deacetylsessein<br>19-hydroxy-7α-acetoxyroyleanone	[100]

	19-acetoxy-7α-acetoxyroyleanone	
S. sahendica Boiss. & Buhse	1,4-dihydro-6-methyl-2-(1-methylethyl)-5-(4-methylpent-4-enyl)naphthalene-1,4-dione<br>sahandinone, sahandone, prionitin, horminone	[101]
S. sahendica Boiss. & Buhse	nor-ambreinolide-18,6α-olide<br>8α-acetoxy-13,14,15,16-tetranorlabdan-12-oic acid-18,6α-olide	[102]
S. sahendica Boiss. & Buhse	salvileucolide methylester<br>8α-hydroxy-13-hydroperoxylabd-14,17-dien-19,16;23,6α-diolide<br>salvileucolide-6,23-lactone<br>17,18,19,20-tetra-nor-13-epi-manoyloxide-14-en-16-oic acid-23,6α-olide	[102, 103]
S. sclarea L.	sclareol, 2,3-dehydrosalvipisone,<br>7-oxoferruginol-18-al	[104]
S. sharifii Rech.f. & Esfand.	ent-13-epi-manoyloxide	[105]
S.willeana (Holmboe) Hedge	carnosic acid, isorosmanol	[59]
**S1.6.3. Triterpenes**		
S. aegyptiaca L.	lupeol, β-amyrin, 3α-hydroxy-24-alkylcarboxylate-12-oleanan-28-oicacid	[106]
S. albocaerulea Lindl.	ursolic acid, 2α-hydroxyursolic acid, maslinic acid	[84]
S. bicolor Lam.	β-amyrin, lupeol	[107]
S. chinensis Benth.	α-boswellic acid	[36]
S. digitaloides Diels	oleanolic acid, glutinol, colchiside A, stachlic acid C	[4]
S. forskahlei L.	α-amyrin	[90]
S. nicolsoniana Ramamoorthy	ursolic acid, oleanolic acid, betulinic acid,<br>3α-24-dihydroxy-olean-12-en-28-oic acid,<br>3α-24-dihydroxy-olean-12-en-28,30-dioic acid	[108]
S. officinalis L.	ursolic acid, oleanolic acid	[72]
S. palaestina Benth.	ursolic acid, oleanolic acid, lupeol,<br>lup-20(29)-ene-2α,3β-diol, lup-20(29)-ene-3β,23-diol,<br>2α-methoxylup-20(29)-en-3β-ol	[96]
S. pratensis L.	β-amyrin, lupeol, loranthol, germanicol	[109]
S. scapiformis Hance	ursolic acid, 4-epi-niga-ichigoside F1<br>niga-ichigoside F1	[42]
S. virgata Jacq.	ursolicacid, oleanolicacid, maslinic acid<br>2α-hydroxy-ursolic acid<br>2α,3α-dihydroxyolean-12-en-28-oic acid<br>2α,3α,23-trihydroxy-olean-12-en-28-oic acid	[59]
S. willeana (Holmboe) Hedge	ursolic acid, oleanolic acid, crataegolicacid<br>2α,3α-dihydroxyolean-12-en-28-oic acid<br>urs-12-ene-3β,11α-diol, urs-12-ene-3α,11α-diol<br>olean-12-ene-3β,11α-diol<br><br>oleanolic acid, lupeol	[59]<br><br><br><br><br>[110]

**S1.7. Abietane diterpene alkaloids**		
S. yunnanensis C.H.Wright	salviamines A-F, isoalviamines C-E	[36]
**S1.8. Apocarotenoids**		
S. nemorosa L.	salvionosides A- C, blumeol C glucoside, (6*S*,9*R*)-reseoside, (6*S*,9*S*)-reseoside	[111]
S. officinalis L.	(6*R*,9*S*)-3-Oxo-α-ionol β-D-glucopyranoside (6*R*,9*R*)-3-Oxo-α-ionol β-D-glucopyranoside	[16]
S. virgata Jacq.	blumenol A	[59]

1. Topcu, G.; Ulubelen, A.; Tam, T.C.-M.; Tao-Che, C. Sesterterpenes and other constituents of *Salvia yosgadensis*. *Phytochemistry* **1996**, *42*, 1089–1092.
2. Zhang, H.-J.; Li, L.-N. Salvianolic acid H, a new depside from *Salvia cavalaries* var. *simplicifolia*. *Chin. Chem. Lett.* **1993**, *4*, 501–504.
3. Zhang, H.-J.; Li, L.-N. Salvianolic acid I, a new depside from *Salvia cavaleriei*. *Planta Med*. **1994**, *60*, 70–72.
4. Wu S. and Chan Y. Five new iridoids from roots of *Salvia digitaloides. Molecules* **2014**, *19*, 15521-15534.
5. Zhao, L.M.; He, W.Y.; Liang, X.T.; Li, L.N. Salviaflaside and salviaflaside methyl ester two new depsidic glycosides from *Salvia flava*. *Chin. Chem. Lett*., **1996a**, *7*, 449–452.
6. Ai, C.B.; Deng, Q.H.; Song, W.Z.; Li, L.N. Salvianolic acid J, a depside from *Salvia flava*. *Phytochemistry* **1994**, *37*, 907–908.
7. Ai, C.-B.; Li, L.-N. Salvianolic acids D and E, Two new depsides from *Salvia miltiorrhiza*. *Planta Med.* **1992**, *58*, 197–199.
8. Yang, C.; Zhang, B. Extraction and isolation of water-solubleactive constituent, Dan Shen Su, from *Salvia miltiorrhiza* and preparationof injections. *Yaoxue Tongbao* **1981**, *16*, 646–647. (Chemical Abstracts 96, 223077p).
9. Kang, H.S.; Chung, H.Y.; Jung, J.H.; Kang, S.S.; Choi, J.S. 1997.Antioxidant effect of *Salvia miltiorrhiza*. *Archives of Pharmacol.Research* **1997**, *20*, 496–500.
10. Kohda, H.; Takeda, O.; Tanaka, S.; Yamasaki, K.; Yamashita, A.; Kurokawa, T.; Ishibashi, S. Isolation of inhibitors of adenylatecyclase from Dan-shen, the root of *Salvia miltiorrhiza*. *Chem. Pharm. Bull.* **1989**, *37*, 1287–1290.
11. Li, L.N.; Tan, R.; Chen, W.M. Salvianolic acid A, a new depsidefrom roots of *Salvia miltiorrhiza*. *Planta Medica* **1984**, *50*, 227–228.
12. Ai, C.B.; Li, L.N. Stereostructure of salvianolic acid B and isolation of salvianolic acid C from *Salvia miltiorrhiza*. *J. Nat. Prod*. **1988**, *51*, 145–149.
13. Ai, C.B.; Li, L.N. Salvianolic acid G, a caffeic acid dimer with a novel tetracyclic skeleton. *Chin. Chem. Lett*. **1991**, *2*, 17–18.
14. Ai, C.-B.; Li, L.-N. Synthesis of tetramethyl salvianolic acid F and trimethyl przewalskinic acid A. *Chin. Chem. Lett*. **1996**, *7*, 427–430.
15. Lu Y.; Foo L.Y. Rosmarinic acid derivatives from *Salvia officinalis*. *Phytochemistry* **1999**, *51*, 91–94.
16. Wang M.; Shao Y.; Huang T.; Wei G.; Ho C. Isolation and structural elucidation of aroma constituents bound as glucosides from Sage (*Salvia officinalis*). *J. Agric. Food Chem*. **1998**, *46*, 2509-2511.
17. Wang, M. ; Shao, Y. ; Li, J. ; Zhu, N. ; Rangarajan, M. ; La Voie, E. ; Ho, C.-T. Antioxidative phenolic glycosides from sage (*Salvia officinalis*). *Journal of Natural Products* **1999**, *62*, 454–456.
18. Generalić I.; Skroza D.; Šurjaka J.; Možinab S.; Ljubenkovc I.; Katalinić A.; Šimate V. and KatalinićV. Seasonal Variations of Phenolic Compounds and Biological Properties in Sage (*Salvia officinalis* L.). *Chem. Biodiv*. **2012**, *9*, 441-456.

19. Lu Y & Foo L.Y. Antioxidant activities of polyphenols from sage (*Salvia officinalis*). *Food Chem.* **2001**, *75*, 197-202.
20. Gong Z.; Ju A.; Zhou D.; Li D.; Zhou W.; Geng W.; Li B.; Li L.; Liu Y.; He Y.; Song M.; Wang Y.; Ye Z.; Lin R. Salvianolic Acid Y, A New Protector of PC12 Cells against Hydrogen Peroxide-Induced Injury from *Salvia officinalis*. *Molecules* **2015**, *20*, 683-692.
21. Wang, M.; Kikuzaki, H.; Zhu, N.; Sang, S.; Nakatani, N.; Ho, C.-T. Isolation and structural elucidation of two new glycosides from sage (*Salvia officinalis*L.). *Journal of Agricultural and FoodChemistry* **2000**, *48*, 235–238.
22. Cuvelier, M.E.; Richard, H.; Berset, C. 1996. Antioxidative activity and phenolic composition of pilot-plant and commercial extracts of sage and rosemary. *J. Am. Oil Chem. Soc.* **1996**, *73*, 645–652.
23. Jerković I.; Mastelić J.; Marijanović Z. A variety of volatile compounds as Markers in Unifloral Honey from Dalmatian Sage (*Salvia officinalis* L.). *Chem. Biodiv*. **2006**, *3*, 1307-1316.
24. Zhao, L.M.; Liang, X.T.; Li, L.N. Prionitisides A and B, twophenolic glycosides from *Salvia prionitis*. *Phytochemistry,* **1996b**, *42*, 899–901.
25. Lu X.Z.; Xi W.H.; Shen J.X.; Naoki, H. Przewalskinic acid A, a new phenolic acid from *Salvia przewalskii* Maxim. *Chin. Chem. Lett.* **1991**, *2*, 301–302.
26. Wu, Z.; Ouyang, M.; Yang, C. Polyphenolic constituents of *Salvia przewalskii*. *Yunnan Zhiwu Yanjiu* **1999**, *21*, 512–516. (ChemicalAbstracts 132, 332013x).
27. Rauter A.; Dias C.; Martins A.; Branco I.; Neng N.; Nogueira J.; Goulart M.; Silva F.; Justino J.; Trevitt C.; Waltho J. Non-toxic *Salvia sclareoides* Brot.extracts as a source of functional food ingredients, Phenolic profile, antioxidant activity and prion binding properties. *Food Chem*. **2012**, *132*, 1930–1935.
28. Wu, Z.; Ouyang, M.; Yang, C. Polyphenolic constituents of *Salvia sonchifolia*. *Yunnan Zhiwu Yanjiu* **1999**, *21*, 393–398. (Chemical Abstracts 132, 205422e).
29. Tanaka, T.; Nishimura, A.; Kouno, I.; Nonaka, G.; Young, T.-J. Isolation and characterization of yunnaneic acids A-D, fournovel caffeic acid metabolites from *Salvia yunnanensis*. *J. Nat. Prod*. **1996**, *59*, 843–849.
30. Tanaka, T.; Nishimura, A.; Kouno, I.; Nonaka, G.; Yang, C.-R. Four new caffeic acid metabolites, yunnaneic acids E-H, from *Salvia yunnanensis*. *Chem. Pharm. Bull*. **1997**, *45*, 1596–1600.
31. El-Missiry, M.M.; Hussiney, H.A.; Ismail, S.I.; Radwan, H.M.; Rizk, A.M. Constituents of plants growing in Qatar XXIV.Phytochemicalinvestigation of *Salvia aegyptiaca* L. *Qatar University Science Journal* **1994**, *14*, 249–251. (Chemical Abstracts 124, 337868z).
32. Yeşilyurt V.; Halfon B.; Öztürk M.; Topçu G. Antioxidant potential and phenolic constituents of *Salvia cedronella*. *Food Chem.* **2008**, *108*, 31–39.
33. Ulubelen, A.; Tuzlaci, E. Flavonoids and triterpenoids from *Salvia euphratica* and *S. longipedicellata*. *Fitoterapia* **1990**, *61*, 185.
34. Lu Y.; Foo L.Y.; Wong, H. Sagecoumarin, a novel caffeic acid trimer from *Salvia officinalis*. *Phytochemistry* **1999**, *52*, 1149–1152.
35. Lu Y.; Foo L.Y. Flavonoid and phenolic glycosides from *Salvia officinalis*. *Phytochemistry* **2000**, *55*, 263–267.
36. Li M.; Li Q.; Zhang C.; Zhang N.; Cui Z.; Huang L.; Xiao P. An ethnopharmacological investigation of medicinal *Salvia* plants (Lamiaceae) in China. *Phytochemistry* **2013**, *59*, 117-140.
37. Wang, M.; Li, J.; Rangarajan, M.; Shao, Y.; La Voie, E.J.; Huang, T.- C.; Ho, C.-T. Antioxidative phenolic compounds from sage (*Salvia officinalis*). *J. Agric. Food Chem*. **1998**, *46*, 4869–4873.
38. Powell, R.G.; Plattner, R.D. Structure of a secoisolariciresinoldiester from *Salvia plebeia* seed. *Phytochemistry* **1976**, *15*, 1963–1965.
39. Plattner, R.D.; Powell, R.G. A secoisolaricireinol branchedfatty diester from *Salvia plebeia* seed. *Phytochemistry* **1978**, *17*, 149–150.

40. Mehmood S.; Riaz N.; Ahmad Z.; Afza N.; Malik A. Lipoxygenase Inhibitory Lignans from *Salvia santolinifolia*. *Pol. J. Chem* **2008**, *82*, 571-575.
41. Mehmood S.; Fatima I. and Malik A. Salvicins A and B, new lignans from *Salvia santolinifolia*. *J Asian Nat Prod Res*. **2011**, *13*, 588–591.
42. Lai L.; Ding S.; Qian H.; Zhang J.; Xue Y.; Luo Z.; Yao G. and Zhang Y. A New Lignan Glucoside from the Whole Plants of *Salvia scapiformis*. *Molecules* **2013**, *18*, 11377-11383.
43. Lu Y.; Foo L.Y. Polyphenolics of Salvia-a review. *Phytochemistry* **2002**, *59*, 117-140.
44. Bisio, A.; Romussi, G.; Ciarallo, G.; De Tommasi, N. Flavonoide und Triterpenoide aus *Salvia blepharophylla* Brandegee ex. Epling. *Pharmazie* **1997**, *52*, 330–331.
45. Topçu, G.; Tan, N.; Ulubelen, A.; Sun, D.; Watson, W.H.Terpenoids and flavonoids from the aerial parts of *Salvia candidssima*. *Phytochemistry* **1995**, *40*, 501–504.
46. Gonzalez, A.G.; Herrera, J.R.; Luis, J.G.; Ravelo, A.G.; Ferro, E.A. Terpenes and flavones of *Salvia cardiophylla*. *Phytochemistry* **1988**, *27*, 1540–1541.
47. Wollenweber, E.; Dörr, M.; Rustaiyan, A.; Roitman, J.N.; Graven, E.H. Exudate flavonoids of some *Salvia* and a *Trichostema* species. *Z. Naturforsch*. **1992**, *47c*, 782–784.
48. Tomás-Lorente, F.; Garcia-Grau, M.; Tomas-Barberan, F. The waste of the industrial treatment of *Salvia lavandulifolia* as a source of biologically active flavonoids. *Fitoterapia* **1998**, *59*, 62–64.
49. Pereda-Miranda, R.; Delgado, G. Flavonoids from *Salvia nicolsoniana*. *J. Nat. Prod.* **1986**, *49*, 1160–1161.
50. Brieskorn, C.H.; Biechele, W. Flavones from *Salvia officinalis*. Compounds of *Salvia officinalis*. *Archiv der Pharmazie (Weinheim)* **1971**, *304*, 557–561.
51. Brieskorn, C.H.; Kapadia, Z. Constituents of *Salvia officinalis*.XXIII, 5-Methoxysalvigenin in leaves of *Salvia officinalis*. *Planta Med*. **1979**,*35*, 376–378.
52. Miski, M.; Ulubelen, A.; Johansson, C. Antibacterial activity studies of flavonoids from *Salvia palaestina*. *J. Nat. Prod*. **1983**, *46*, 874–875.
53. Jiang, Y.; Luo, S.; Zheng, M. Active principles of *Salvia plebeia*. *Yiyao Gongye* **1987**, *18*, 349–351. (Chemical Abstracts 107, 233142r).
54. Oshima, Y.; Kawakami, Y.; Kiso, Y.; Hikino, H.; Yang, L.L.; Yen, K.Y. Liver protective drugs. 13. Antihepatotoxic principles of *Salvia plebeia* herbs. *Shoyakugaku Zasshi* **1984**, *38*, 201–202. (Chemical Abstracts 102, 56094a).
55. Gupta, H.C.; Ayengar, K.N.N.; Rangaswami, S. Structure and synthesis of salvitin, a new flavone isolated from *Salvia plebeia*. *Ind. J. Chem*. **1975**, *13*, 215–217.
56. Ulubelen, A.; Ozturk, S.; Isildatici, S. A new flavone from *Salvia triloba*. *J. Pharm. Sci*. **1968**, *57*, 1037–1038.
57. Abdalla, M.F.; Saleh, N.A.M.; Gabr, S.; Abu-Eyta, A.M.; El-Said, H. Flavone glycosides of *Salvia triloba*. *Phytochemistry* **1983**, *22*, 2057–2060.
58. Ulubelen, A.; Ayanoglu, E. Flavonoids of *Salvia virgata*. *Lloydia* **1975**, *38*, 446–447.
59. De la Torre M.; Bruno M.; Savona F.; Rodriquez B.; Apostolides Arnold N. Terpenoids from *Salvia willeana* and *S.* virgata. *Phytochemistry* **1990**, *29*, 668-670.
60. Chen, C.C.; Chen, H.T.; Chen, Y.P.; Hsu, H.Y.; Hsieh, T.C. Isolation of the components of *Salviae miltiorrhizae* radix and their coronary dilator activities. *Taiwan Yao Hsueh Tsa Chih* **1986**, *38*, 226–230. (Chemical Abstracts 107, 211619z).
61. Gonzalez, A.G.; Aguiar, Z.E.; Luis, J.G.; Ravelo, A.G.; Vazquez, J.T.; Dominquez, X.A. Flavonoids from *Salvia texana*. *Phytochemistry* **1989**, *28*, 2871–2872.
62. Gökdil, G.; Topçu, G.; Sönmez, U.; Ulubelen, A. Terpenoids and flavonoids from *Salvia cyanescens*. *Phytochemistry* **1997**, *46*, 799–800.
63. Wollenweber, E. Flavones and flavonoids in exudate of *Salvia glutinosa*. *Phytochemistry* **1974**, *13*, 753.
64. Abdalla, M.F. The flavonoids of some local *Salvia* species. *Egyptian Journal of Chemistry* **1984**, *27*, 827–829.

65. Zhao, L.; Liang, X.; Li, L. Two minor phenolic glycoside from *Salvia cavaleriei*. *J. Chin. Pharm. Sci.* **1997**, *6*, 111–112.
66. Tomás-Barberàn, F.A.; Harborne, J.B.; Self, R. Dimalonated anthocyanins from the flowers of *Salvia splendens* and *S. coccinea*. *Phytochemistry* **1987**, *26*, 2759–2760.
67. Kamel, M.S.; Desoky, E.K.; Abdallah, O.M.; Bishay, D.W. Flavonol glycosides from leaves of *Salvia farinacea* Benth. *Bull. Fac. Pharm.* (Cairo University) **1992**, *30*, 259–262. (ChemicalAbstracts 119, 156237u).
68. Kondo, T.; Yoshikane, M.; Yoshida, K.; Goto, T. Structure of anthocyanins in scarlet, purple, and blue flowers of *Salvia*. *Tetrahedron Lett*. **1989**, *30*, 6729–6732.
69. Kokkalou, E.; Kapetanidis, I. Flavonoides et acides phenoliques de *Salvia horminum* L. (Lamiaceae). *Pharm.Acta Helv*. **1988**, *63*, 90–92.
70. Canigueral, S.; Iglesias, J.; Hamburger, M.; Hostettmann, K. Phenolic constituents of *Salvia lavandulifolia* ssp. *lavandulifolia*. *Planta Med.* **1989**, *55*, 92.
71. Zarzuelo, A.; Gamez, J.M.; Utrilla, P.; Jimenez, J.; Jimenez, I. Luteolin 5-rutinoside from *Salvia lavandulifolia* ssp. *oxyodon*. *Phytochemistry* **1995**, *40*, 1321–1322.
72. Mašterová, I.; Uhrín, D.; Kettmann, V.; Suchý, V. Phytochemical study of *Salvia officinalis* L. *Chem. Pap*. **1989**, *43*, 797–803. (Chemical Abstracts 112, 731917v).
73. Takeda, K.; Yanagisawa, M.; Kifune, T.; Kinoshita, T.; Timberlake,C.F. A blue pigment complex in flowers of *Salvia patens*. *Phytochemistry* **1994**, 35, 1167–1169.
74. Smirnova, L.P.; Glyzin, V.I.; Patudin, A.V.; Ban'kovskii, A.I. Flavones from some *Salvia* species. *Khim. Prir. Soedin*. **1974b**, *10*, 668–669. (Chemical Abstracts 82, 83016b).
75. Asen, S. Anthocyanins in flowers of *Salvia splendens* cultivar *violet* Flame. *Proceedings Am Soc Hortic Sci*. **1961**, *78*, 586–592.
76. Shibata, M.; Uragami, S.; Matsuura, K. Paper-chromatographic survey of anthocyanins in purple Salvia flowers. *Bot. Magaz*. (Tokyo) **1966**, *79*, 537–543.
77. Veitch, N.C.; Grayer, R.J.; Irwin, J.L.; Takeda, K. Flavonoid cellobiosides from *Salvia uliginosa*. *Phytochemistry* **1998**, *48*, 389–393.
78. Ishikawa T.; Kondo T.; Kinoshita T.; Haruyama H.; Inaba S.; Takeda K.; Grayer R.J.; Veitch N.C. An acetylated anthocyanin from the blue petals of *Salvia uliginosa*. *Phytochemistry* **1999**, *52*, 517–521.
79. Saleh, M.R.I.; Sabri, N.N. Phytochemical study of the herb, *Salvia verbenaca*. *J. Drug Res*. **1980**, *12*, 131–137.
80. Ulubelen A.; Topçu G. Flavonoids and Terpenoids From *Salvia verticillata* and *Salvia pinnata*. *J. Nat. Prod.* **1984**, *47*, 1068–1068.
81. Miura K.; Kikuzaki H.; Nakatani N. Apianane terpenoids from *Salvia officinalis*. *Phytochemistry* **2001**, *58*, 1171-1175.
82. Lu X.; Xu W.; Naoki H. Anthraquinones from *Salvia przewalskii*. *Phytochemistry* **1992**, *31*, 708-709.
83. Cao S.; Ke Z.; Xi L. A new sesquiterpene lactone from *Salvia plebeian*. *J. Asian Nat. Prod. Res*. **2013**, *15*, 404-407.
84. Pereda-Miranda, R.; Hernández L.; López, R. A Novel Antimicrobial Abietanetype Diterpene from *Salvia albocaerulea*. *Planta Med.* **1992**, *58* , 223–224.
85. Taira Z.; Watson W. The Structure of Conacytone, $C_{20}H_{26}O_6$, a Diterpene Quinone from *Salvia ballotaeflorae*. *Acta Cryst*. **1976**, *32*, 2149.
86. Wu S.; Chan H.; Hwang R.; Qian K.; Morris-Natschke S.; Lee K.; Wu T. Salviatalin A and salvitrijudin A, two diterpenes with novel skeletons from roots of *Salvia digitaloides* and anti-inflammatory evaluation. *Tetrahedron Lett.* **2010**, *51*, 4287-4290.
87. Valdés L. *Salvia divinorum* and the unique diterpene hallucinogen, Salvinorin (Divinorin) A. *J. Psychoactive Drugs* **1994**, *26*, 277-283.
88. Medana C.; Massolino C.; Pazzi M.; Baiocchi C. Determination of salvinorins and divinatorins in *Salvia divinorum* leaves by liquid chromatography/ multistage mass spectrometry. *Rapid Commun Mass Spectrom* **2006**, *20*, 131-136.

89. Ahmed A.; Mohamed A.; Karchesy J.; Asakawa Y. Salvidorol, a nor-abietane diterpene with a rare carbon skeleton and two abietane diterpene derivatives from *Salvia dorrii. Phytochemistry* **2006**, *67*, 424-428.
90. Ulubelen A.; Sönmez U.; Topcu G.; Johansson C. Abierane diterpene and two phenolics from *Salvia forskahlei. Phytochemistry* **1996**, *42*, 145-147.
91. Ulubelen A.; Topcu G.; Tan N. Diterpenoids from *Salvia heldrichiana. Phytochemistry* **1995**, *40*, 1473-1475.
92 Ulubelen, A.; Miski, M.; Neuman, P.; Mabry, T.J. Flavonoids of *Salvia tomentosa* (Labiatae). *J. Nat. Prod*. **1979**, *42*, 261–263.
93. Bautista E.; Maldonado E.; Ortega A. neo-Clerodane Diterpenes from *Salvia herbacea*. *J. Nat. Prod.* **2012**, *75*, 951-958.
94. Chang H.; Cheng K.; Choang T.; Chow H.; Chui K.; Hon P.; Tan F.; Yang Y.; Zhong Z. Structure elucidation and total synthesis of new tanshinones isolated from *Salvia miltiorrhiza* Bunge (Danshen). *J. Org. Chem*. **1990**, *55*, 3537-3543.
95. Tada M.; Hara T.; Hara C.; Chiba K. A quinine methide from *Salvia officinalis. Phytochemistry*, **1997**, *45*, 1475-1477.
96. Santos-Gomes P.; Seabra R.; Andrade P.; Fernandes-Ferreira M. Wu Phenolic antioxidant compounds produced by in vitro shoots of sage (*Salvia officinalis* L.). *Plant Sci.* **2002**, *162*, 981-987.
97. Hussein A.; Torre M.; Rodríquez B.; Hammouda F.; Hussiney H. Modified abietane diterpenoids and a methoxylupane derivative from *Salvia palaestina*. *Phytochemistry* **1997**, *45*, 1663-1668.
98. Shirsat R.; Suradkar S.; Koche D. Some phenolic compounds of *Salvia plebeian R. Br. BD* **2012**, *3*, 61-63.
99. Jiang H.; Wang X.; Xiao J.; Luo X.; Yao X.; Zhao Y.; Chen Y.; Crews P.; Wu Q. New abietane diterpenoids from the roots of *Salvia przewalskii. Tetrahedron* **2013**, *69*, 6687-6692.
100. Hernández M.; Esquivel B.; Cárdenas J.; Rodriguez-Hahn L.; Ramamoorthy T.P. Diterpenoid abietane quinones isolated from *Salvia regla. Phytochemistry 26*, 3297-3299.
101. Jassbi A. R.; Mehrdadb M.; Eghtesadic F.; Ebrahimic S.; Baldwin I. Novel Rearranged Abietane Diterpenoids from the Roots of *Salvia sahendica*. *Chem. Biodiv*. **2006**, *3*, 916-922.
102. Moghaddam F.; Farimani M.; Seirafi M.; Taheri S.; Khavasi H.; Sendker J.; Proksch P.; Wray V.; Edrada R. Sesterterpenoids and other constituents of *Salvia sahendica. J. Nat. Prod.* **2010**, *73*, 1601-1605.
103. Moghaddam F.; Zaynizadeh B.; Rüedi P. Salvileucolide methylester, a sesterterpene from *Salvia sahendica. Phytochemistry* **1995**, *39*, 715-716.
104. Ulubelen A.; Topcu G.; Eriş C.; Sönmez U.; Kartal M.; Kurucu S.; Johansson C. Terpenoids from *Salvia sclarea*. *Phytochemistry* **1994**, *36*, 971-974.
105. Farjam M.; Rustaiyan A.; Ezzatzadeh E.; Jassbi A. Labdane-Type Diterpene and Two Flavones from *Salvia Sharifii* Rech.f. and Esfan. and their Biological Activities. *Iran J Pharm Res.* **2013**, *12*, 395-399.
106. Basaif S. Chemical constituents of *Salvia aegyptiaca. JKAU* **2004**, *16*, 33-39.
107. Ibrahim T. Chemical composition and biological activity of extracts from *Salvia bicolor* Desf. growing in Egypt. *Molecules* **2012**, *17*, 11315-11334.
108. Delgado, G.; Vivar, A. New triterpenoids from *Salvia nicolsoniana*. *J. Nat. Prod.* **1986**, *49*, 225-230.
109. Anaya, J.; Caballero, M.C.; Grande, M.; Navarro, J.J.; Tapia, I.; Almeida, J.F. A lupeol derivative from *Salvia pratensis*. *Phytochemistry* **1989**, *28*, 2206-2208.
110. Vonaparti A.; Karioti A.; Recio M.; Máñez S.; Ríos J.; Skaltsa E.; Giner R. Effects of terpenoids from *Salvia willeana* in delayed-type hypersensitivity, human lymphocyte proliferation and cytokine production. *Nat. Prod. Comm.* **2008**, *3*, 1953-1958.

111. Takeda, Y.; Zhang, H.; Matsumoto, T.; Otsuka, H.; Oosio, Y.; Honda, G.; Tabata M.; Fujita T.; Sun H.; Sezik E.; Yesilada E. Megastigmane glycosides from *Salvia nemorosa. Phytochemistry* **1997**, *44*, 117-120.
